# A Loop‐Based and AGO‐Incorporated Virtual Screening Model Targeting AGO‐Mediated miRNA–mRNA Interactions for Drug Discovery to Rescue Bone Phenotype in Genetically Modified Mice

**DOI:** 10.1002/advs.201903451

**Published:** 2020-05-28

**Authors:** Zhenjian Zhuo, Youyang Wan, Daogang Guan, Shuaijian Ni, Luyao Wang, Zongkang Zhang, Jin Liu, Chao Liang, Yuanyuan Yu, Aiping Lu, Ge Zhang, Bao‐Ting Zhang

**Affiliations:** ^1^ School of Chinese Medicine Faculty of Medicine The Chinese University of Hong Kong Hong Kong SAR China; ^2^ Aptacure Therapeutics Limited Kowloon Hong Kong SAR China; ^3^ Law Sau Fai Institute for Advancing Translational Medicine in Bone & Joint Diseases School of Chinese Medicine Hong Kong Baptist University Hong Kong SAR China; ^4^ Institute of Integrated Bioinformedicine and Translational Science School of Chinese Medicine Hong Kong Baptist University Hong Kong SAR China; ^5^ Guangdong‐Hong Kong‐Macao Greater Bay Area International Research Platform for Aptamer‐based Translational Medicine and Drug Discovery Hong Kong 999077 China; ^6^ Department of Biochemistry and Molecular Biology School of Basic Medical Sciences Southern Medical University Guangdong Provincial Key Laboratory of Single Cell Technology and Application Guangzhou 510515 China

**Keywords:** bone rescue, drug discovery, miRNA–mRNA interactions, small molecule screening, virtual screening models

## Abstract

Several virtual screening models are proposed to screen small molecules only targeting primary miRNAs without selectivity. Few attempts have been made to develop virtual screening strategies for discovering small molecules targeting mature miRNAs. Mature miRNAs and their specific target mRNA can form unique functional loops during argonaute (AGO)‐mediated miRNA–mRNA interactions, which may serve as potential targets for small‐molecule drug discovery. Thus, a loop‐based and AGO‐incorporated virtual screening model is constructed for targeting the loops. The previously published studies have found that miR‐214 can target ATF4 to inhibit osteoblastic bone formation, whereas miR‐214 can target TRAF3 to promote osteoclast activity. By using the virtual model, the top ten candidate small molecules targeting miR‐214‐ATF4 mRNA interactions and top ten candidate small molecules targeting miR‐214‐TRAF3 mRNA interactions are selected, respectively. Based on both in vitro and in vivo data, one small molecule can target miR‐214‐ATF4 mRNA to promote ATF4 protein expression and enhance osteogenic potential, whereas one small molecule can target miR‐214‐TRAF3 mRNA to promote TRAF3 protein expression and inhibit osteoclast activity. These data indicate that the loop‐based and AGO‐incorporated virtual screening model can help to obtain small molecules specifically targeting miRNA–mRNA interactions to rescue bone phenotype in genetically modified mice.

## Graphical abstract

It is found that mature miRNA and their specific target mRNA can form unique functional loops during argonaute (AGO)‐mediated miRNA–mRNA interactions. Thus, a loop‐based and AGO‐incorporated virtual screening model is constructed for screening small molecules targeting the loops. By using the virtual model, two small molecules are obtained that can specifically target miR‐214‐ATF4 mRNA interactions and miR‐214‐TRAF3 mRNA interactions, respectively.

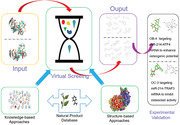

## 1 Introduction

MicroRNAs (miRNAs) are endogenous, single‐stranded and non‐coding RNAs, which regulate hundreds of genes and play key roles in a number of physiological and pathological processes, such as proliferation, differentiation, and apoptosis.^[^ ^1^ ^]^ Recently, an innovative approach Inforna has been reported, which enables the rational design of small molecules targeting RNA.^[^ ^2^ ^]^ Using the Inforna, a small molecule specially binding to primary microRNA‐96 with high affinity was identified.^[^ ^2c^ ^]^ However, this strategy to target primary miRNAs lacks selectivity because one miRNA might regulate various target mRNAs, thus regulate various biological processes. Based on the Inforna, it is desirable for drug discovery to develop a selective small molecule strategy that could specifically target the interaction between miRNA and its target mRNA to rescue the inhibition of mRNA translation.

It is known that miRNAs guide the miRNA‐induced silencing complexes (miRISCs) to the 3′‐untranslated regions (3′‐UTR) of target mRNAs through base complementarity paring.^[^ ^3^ ^]^ Argonaute (AGO) protein, core component of miRISCs, then cleaves, destabilizes, or translationally inhibits mRNAs to modulate physiological and pathological processes.^[^ ^4^ ^]^ Previous studies have elucidated that, in mammals, specific miRNAs and their target mRNAs could form unique structures which can be called as loops.^[^ ^5^ ^]^ The AGO protein contains a PAZ domain which is involved in miRNA, and a PIWI domain which is related to RNaseH endonucleases and functions in slicer activity.^[^ ^6^ ^]^ miRNA–mRNA loops mainly distributed near the “seed” region, where AGO exerts its function. The change of the conformation of miRNA–mRNA may lead to the change of cleavage activity of AGO.^[^ ^7^ ^]^ Specific miRNAs and their target mRNAs could form unique structures, which make them promising targets for developing therapeutics in specific diseases.^[^ ^5^ ^]^ Our study further suggested the structural uniqueness, functional importance, high stability, and high specificity of the loops formed by mature miRNA and their specific target mRNA. Therefore, a loop formed by miRNA and its target mRNA could be postulated as a target for drug discovery to specifically target the interaction between miRNA and its target mRNA to rescue the inhibition of mRNA translation. Moreover, as the change of conformation of miRNA–mRNA may lead to the change of cleavage activity of AGO, we further calculated the energy change of the AGO–miRNA–mRNA complex before and after binding of the candidate small molecules in our model to enhance the hit rate.

So, we constructed a loop‐based and AGO‐incorporated virtual screening model targeting AGO‐mediated miRNA–mRNA interaction for drug discovery. Among those miRNAs involved in bone disease, miR‐214 ranks as one of the most studied and elucidated miRNAs. miR‐214 is highly conservative among vertebrates.^[^ ^8^ ^]^ It is known that ATF4 protein is a transcription factor responsible for promoting osteogenic differentiation and osteoblastic function.^[^ ^9^ ^]^ Our previous study first identified that miR‐214 could target ATF4 mRNA to inhibit bone formation.^[^ ^9^ ^]^ Several researches have reported that miR‐214 is also capable of enhancing osteoclast differentiation. In our previous study, we found that miR‐214 was significantly increased in bone specimens from breast cancer patients with osteolytic bone metastasis (OBM) as well as in osteoclasts from nude mice with human breast cancer xenografts during OBM development, which was accompanied by the elevated bone resorption.^[^ ^10^ ^]^ TRAF3 is a vital regulator of type I interferons and cytokine production that regulates RANKL‐induced osteoclast formation.^[^ ^11^ ^]^ TRAF3 has a negative regulatory role in osteoclastic precursors. Osteoclastic miR‐214 could target TRAF3 mRNA to promote osteoclast activity. Thus, we screened small‐molecule candidates that could target miR‐214‐ATF4 and miR‐214‐TRAF3, respectively, to rescue bone phenotype in osteoblast‐ and osteoclast‐specific genetic models for validating the usage of our loop‐based and AGO‐incorporated virtual screening strategy.

## 2 Results

### 2.1 Loop, Formed by miRNA and Its Target mRNA, as a Postulated Target

**Figure 1:**
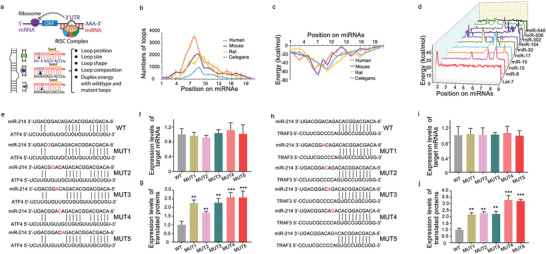
Statistic data for the functional validation of the loops in miRNA‐mRNA interaction. a) The 2D and 3D of the loops in miRNA–mRNA interaction, respectively. b) The statistic distribution of the numbers of the loops in miRNA–mRNA interactions among distinct species. c) The statistic distribution of the energy of the loops in miRNA–mRNA interactions among distinct species. d) The binding position profiles of the miRNA–mRNA loops in the listed miRNA families. e) The wild‐type and mutant loops in miR‐214‐ATF4 interaction. f) The effects of the mutated loops on expression levels of the target ATF4 mRNAs. g) The effects of the mutated loops on levels of ATF4 proteins translated by the target mRNAs. h) The wild‐type and mutant loops in miR‐214‐TRAF3 interaction. i) The effects of the mutated loops on expression levels of the target TRAF3 mRNAs. j) The effects of the mutated loops on levels of TRAF3 proteins translated by the target mRNAs. **p < 0.01, ***p < 0.001 versus WT group.

To investigate the potential of the loop‐formed miRNA and its target mRNA as a postulated target, we first determine the function of the loop. By statistical analysis of miRNA‐mRNA interaction descriptor, it was found that loop formed by miRNAs and mRNAs could be defined by combining the position, energy, size, shape, and base composition to exert their inhibition role in protein expression (**Figure** **1**a). By statistical analysis of prediction of target sites, it was found that the loop (formed by mature miRNA and their specific target mRNA) mainly distributed near the seed region (position 1–10) of the mature miRNA (the key region for binding to the target mRNA) among distinct species (Figure 1b), implying that the distribution of the loops in miRNA–mRNA interactions could be highly conserved among distinct species. By statistical analysis of calculation of minimum free energy to analyze the predicted energy of the mutated loops in miRNA–mRNA interactions among distinct species, it was found that the mutation consistently induced the lower predicted energy of the miRNA–mRNA complex near the seed region (position 1–10) of the mature miRNA, implying the high stability of the complex after targeting the loops (Figure 1c). By statistical analysis of calculation of the profiles of miRNA–mRNA loops in different miRNA families, it was found that the binding position profile of miRNA–mRNA loops among the listed miRNA families were distinctive (Figure 1d). Our previously published studies found that miR‐214 could target ATF4^[^ ^9^ ^]^ and TRAF3^[^ ^10^ ^]^ to inhibit osteoblastic bone formation and promote osteoclast activity, respectively. So, the loop formed by miR‐214‐ATF4 mRNA interaction (Figure 1e) and the loop formed by miR‐214‐TRAF3 mRNA interaction (Figure 1h) were further mutated, respectively. The expression levels of the target mRNAs were not altered (Figure 1f,i), whereas the expression levels of the corresponding proteins translated from the target mRNAs were elevated (Figure 1g,j). Furthermore, ten most widely validated miRNA–mRNA interactions in meta‐analysis based on PubMed library were selected (Tables S1 and S2, Supporting Information). The point mutations were performed on the loops. Consistently, the expression levels of the target mRNAs were not altered, whereas the expression levels of the corresponding proteins translated from the target mRNAs were elevated (Figure S1, Supporting Information). Taken together, the above data indicated that loop formed by miRNA and its target mRNA could be postulated as a target.

To compare the difference in the predicted miRNA–mRNA secondary structure by different prediction methods, RNAfold, Mfold, and AveRNA were adopted, respectively. Five miRNA–mRNA sequences were randomly selected for prediction. The secondary structures of miRmR_1 predicted by Mfold had a little difference compared with RNAfold and AveRNA, whose predicted structures were almost the same for all the selected miRNA–mRNA sequences. Together, the prediction data indicated no obvious difference in the predicted miRNA–mRNA secondary structures among the three adopted prediction methods (Table S3, Supporting Information).

To verify the reasonability that miRNA–mRNA interaction sequence (forming loops) could be input as a single RNA sequence to predict secondary structure, RNAfold was adopted to compare the difference in the predicted secondary structures of “a single miRNA–mRNA interaction sequence” and “a miRNA‐placeholders‐mRNA sequence that miRNA and mRNA were divided by random placeholders on their ligation site.” The data demonstrated that the predicted secondary structures of the two different sequences (a single miRNA–mRNA interaction sequence and a miRNA‐placeholders‐mRNA sequence) were almost the same, where the single miRNA–mRNA interaction sequence was hardly influenced by the inserted random placeholders (Table S4, Supporting Information). It indicated that the miRNA–mRNA interaction feature calculated as a single RNA sequence from 5′ to 3′ could be reasonable.

The features derived from the characterization of miRNA–mRNA–small molecule complex were ranked according to their variable importance measures to the trained model.^[^ ^12^ ^]^ The data showed that the features of miRNA–mRNA interactions took up 54.3% effect on the prediction of the model (feature importance and ranking.xlsx, https://github.com/wanyy063700/SMTRS). To figure out the specificity of loops, the top 143 variable importance measures above 0.001 could be considered that had effect on the specificity of miRNA–mRNA loops to a certain extent, which took up nearly 98% importance measures of miRNA–mRNA feature set. The bottom 37 variable importance measures were 0, which could be considered that had little effect on the specificity of miRNA–mRNA loops. A small molecule (SMILES: OCC(O)C(O)C(O)C(O)CO) was employed to predict 20 miRNA–mRNA interactions. The top 17 candidates were positive while the others were negative (Table S6, Supporting Information). Their feature distribution conformed our analysis according to the RNA_ID (datafile_refined_cleaned_rna_features.csv, https://github.com/wanyy063700/SMTRS).

### 2.2 Construction, Application, and Merits of a Loop‐Based and AGO‐Incorporated Virtual Screening Model

**Figure 2:**
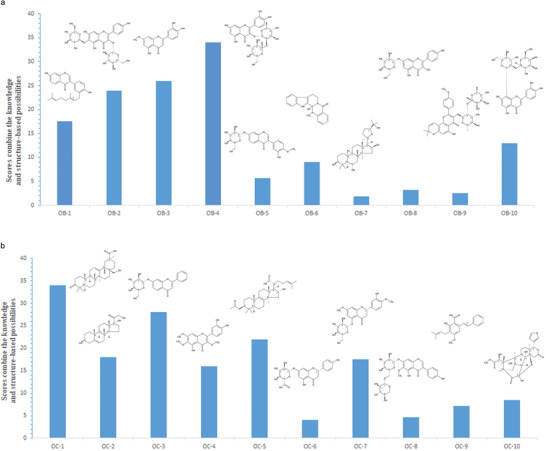
Virtual screening of small‐molecule candidates using virtual screening model score algorithm. a) Top 10 candidate small molecules with the highest score were most likely predicted to target the loop of miR‐214 and ATF4 mRNA. OB‐1: 3′‐geranyl‐4′,7‐dihydroxyisoflavone; OB‐2: 6‐hydroxykaempferol 3,6‐diglucoside; OB‐3: 3′‐hydroxygenkwanin; OB‐4: quercetin‐3‐*O*‐d‐glucosyl]‐(1‐2)‐l‐rhamnoside; OB‐5: 3′,7‐dihydroxy‐4′‐methoxyisoflavone‐7‐beta‐d‐glucopyranoside; OB‐6: hydroxyevodiamine; OB‐7: cycloastragenol; OB‐8: kaempferol‐7‐*O*‐*β*‐d‐glucopyranoside; OB‐9: sutchuenmedin A; OB‐10: 2″‐*O*‐beta‐l‐galactopyranosylorientin. b) Top 10 candidate small molecules with the highest score were most likely predicted to target the loop of miR‐214 and TRAF3 mRNA. OC‐1: 28‐hydroxy‐3‐oxoolean‐12‐en‐29‐oic acid; OC‐2: 17,21‐dihydroxypregnenolone; OC‐3: 7‐hydroxyflavone‐beta‐d‐glucoside; OC‐4: 3,6,7‐trimethylquercetagetin; OC‐5: 3‐*O*‐acetyl‐16alpha‐hydroxytrametenolic acid; OC‐6: naringenin‐7‐*O*‐glucuronide; OC‐7: persicoside; OC‐8: tectorigenin 7‐*O*‐xylosylglucoside; OC‐9: cajaninstilbene acid: OC‐10: 1‐*O*‐deacetyl‐2*α*‐hydroxykhayanolide E.

**Figure 3:**
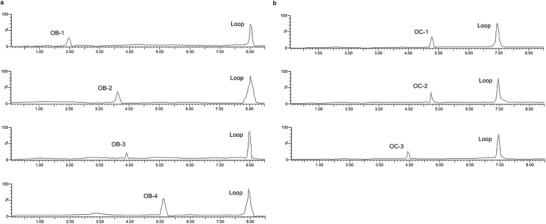
Binding assay of the small‐molecule candidates with the loops. a) Pull‐down assay was performed using streptavidin magnetic bead to examine the interaction between biotin‐miR‐214‐ATF4 loop and small molecules OB‐1, OB‐2, OB‐3, and OB‐4, respectively. Small molecules OB‐1, OB‐2, OB‐3, and OB‐4 captured by biotin‐miR‐214‐ATF4 loop were examined by LC‐MS, respectively. b) Pull‐down assay was performed using streptavidin magnetic bead to examine the interaction between biotin‐miR‐214‐TRAF3 loop and small molecules OC‐1, OC‐2, and OC‐3, respectively. Small molecules OC‐1, OC‐2, and OC‐3 captured by biotin‐miR‐214‐TRAF3 loop were examined by LC‐MS, respectively.

Based on the above structural uniqueness, functional importance, high stability, and high specificity of the loops formed by mature miRNA and their specific target mRNA, a loop‐based and AGO‐incorporated virtual screening model to calculate a list of candidate small molecules targeting the complex of miRNA and its target mRNA was constructed. The loop‐based and AGO‐incorporated virtual screening model can be divided into two calculation algorithms. First, the knowledge‐based machine learning algorithm could facilitate screening an RNA motif–small molecule database to generate a list of candidate small molecules from a natural product database to target the loop (Figure S2a, Supporting Information). In addition, the structure‐based algorithm could calculate the binding energy of AGO‐miRNA‐target mRNA–small molecule complex after docking to generate the other list of candidate small molecules from a natural product database (Figure S2b, Supporting Information). Then, the rankings were combined for the knowledge‐based and structure‐based possibilities to get the top molecule candidates (Figure S2c, Supporting Information). Random forest (RF) model was chosen after comparisons between seven machine learning methods, top ten candidate small molecules (OB‐1: 3′‐geranyl‐4′,7‐dihydroxyisoflavone; OB‐2: 6‐hydroxykaempferol 3,6‐diglucoside; OB‐3: 3′‐hydroxygenkwanin; OB‐4: quercetin‐3‐*O*‐d‐glucosyl]‐(1‐2)‐l‐rhamnoside; OB‐5: 3′,7‐dihydroxy‐4′‐methoxyisoflavone‐7‐beta‐d‐glucopyranoside; OB‐6: hydroxyevodiamine; OB‐7: cycloastragenol; OB‐8: kaempferol‐7‐*O*‐*β*‐d‐glucopyranoside; OB‐9: sutchuenmedin A; OB‐10: 2″‐*O*‐beta‐l‐galactopyranosylorientin) targeting miR‐214‐ATF4 mRNA were selected by the virtual screening model score algorithm (**Figure** **2**a). Similarly, top ten candidate small molecules (OC‐1: 28‐hydroxy‐3‐oxoolean‐12‐en‐29‐oic acid; OC‐2: 17,21‐dihydroxypregnenolone; OC‐3: 7‐hydroxyflavone‐beta‐d‐glucoside; OC‐4: 3,6,7‐trimethylquercetagetin; OC‐5: 3‐o‐acetyl‐16alpha‐hydroxytrametenolic acid; OC‐6: naringenin‐7‐o‐glucuronide; OC‐7: persicoside; OC‐8: tectorigenin 7‐*O*‐xylosylglucoside; OC‐9: cajaninstilbene acid: OC‐10: 1‐*O*‐deacetyl‐2*α*‐hydroxykhayanolide E) targeting miR‐214‐TRAF3 mRNA were selected by the virtual screening model score algorithm (Figure 2b). The biotin‐labeled miR‐214‐ATF4 loop and the biotin‐labeled miR‐214‐TRAF3 loop were synthesized in vitro and incubated with the above top ten small‐molecule candidates (OB‐1 to OB‐10) and the above top ten small molecule candidates (OC‐1 to OC‐10), respectively. After that, streptavidin (SA) beads were added to the mixtures. The liquid chromatography mass spectrometry (LC‐MS) was used to determine the binding ability. Among them, four small molecules OB‐1, OB‐2, OB‐3, OB‐4, and three small molecules OC‐1, OC‐2, OC‐3, could bind to miR‐214‐ATF4 loop and miR‐214‐TRAF3 loop in vitro, respectively, as evidenced by the LC‐MS data (**Figure** **3**a,b). The other six and the other seven small‐molecule candidates could not bind to miR‐214‐ATF4 loop and miR‐214‐TRAF3 loop in vitro, respectively, as evidenced by the LC‐MS data (Figure S3a,b, Supporting Information).

**Figure 4:**
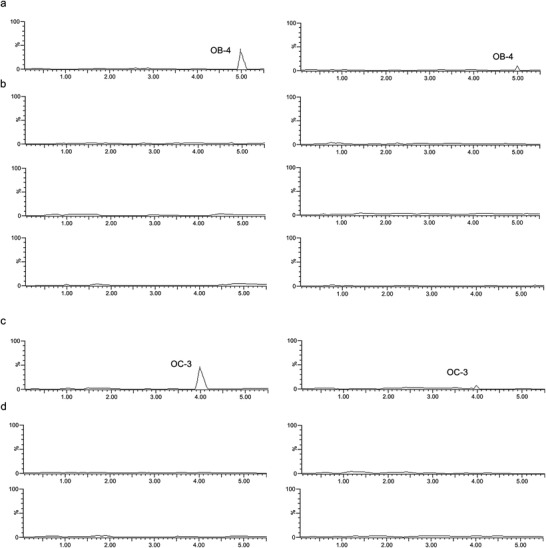
Determination of interaction of the small‐molecule candidates with AGO2. a) The LC‐MS data for the amount of small molecule OB‐4 attached to AGO2 in stably expressing miR‐214 MC3T3‐E1 cells (left), compared to control MC3T3‐E1 cells (right). b) The LC‐MS data for the amount of small molecules OB‐1, OB‐2, and OB‐3 (from top to bottom), respectively, attached to AGO2 in stably expressing miR‐214 MC3T3‐E1 cells (left), compared to control MC3T3‐E1 cells (right). c) The LC‐MS data for the amount of small molecule OC‐3 attached to AGO2 in stably expressing RAW 264.7 cells (left), compared to control RAW 264.7 cells (right). d) The LC‐MS data for the amount of small molecules OC‐1 and OC‐2 (from top to bottom), respectively, attached to AGO2 in stably expressing miR‐214 RAW 264.7 cells (left), compared to control RAW 264.7 cells (right).

MC3T3‐E1 and RAW 264.7 cells stably expressing miR‐214 using lentivirus infection were established. Elevated expression of miR‐214 was confirmed by qRT‐PCR in the miR‐214‐overexpressing MC3T3‐E1 and RAW 264.7 cells (Figure S4a,b, Supporting Information). The miR‐214‐overexpressing MC3T3‐E1 and RAW 264.7 cells were treated with the small molecules OB‐1, OB‐2, OB‐3, OB‐4, and OC‐1, OC‐2, OC‐3, respectively, while the MC3T3‐E1 and RAW 264.7 cells were treated with the small molecules OB‐1, OB‐2, OB‐3, OB‐4, and OC‐1, OC‐2, OC‐3, respectively, as controls. Anti‐AGO2‐small molecule interaction assay was used to determine the potential interaction of the small molecules OB‐1, OB‐2, OB‐3, OB‐4, and OC‐1, OC‐2, OC‐3, with AGO2, respectively. LC‐MS was used to determine the binding ability. The LC‐MS data showed that the amount of the small molecules OB‐4 and OC‐3 attached to AGO2 was higher in miR‐214‐overexpressing MC3T3‐E1 and RAW 264.7 cells, compared to the control MC3T3‐E1 and RAW 264.7 cells (**Figure** **4**a,c). However, there were no differences in the amount of the small molecules OB‐1, OB‐2, and OB‐3, OC‐1 and OC‐2 attached to AGO2 between the miR‐214‐overexpressing MC3T3‐E1 cells and RAW 264.7 and the control MC3T3‐E1 and RAW 264.7 cells, respectively (Figure 4b,d). It was further found that both the small molecules OB‐4 and OC‐3 have high scores in both the knowledge‐based algorithm and the structure‐based algorithm. However, the small molecules OB‐1, OB‐2, OB‐3, OC‐1, and OC‐2 only have high scores in the knowledge‐based algorithm or structure‐based algorithm (Figure S5a,b, Supporting Information).

### 2.3 Determination of Small Molecule OB‐4 That Targets miR‐214‐ATF4 Loop for Rescuing Bone Phenotype in Osteoblast‐Specific miR‐214 Transgenic Mice

**Figure 5:**
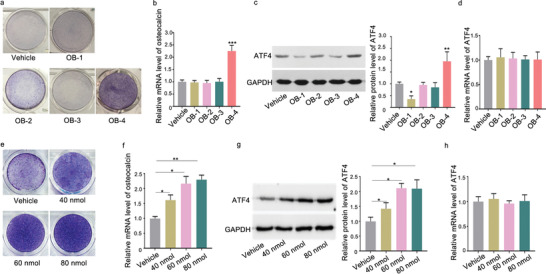
Small molecule OB‐4 could promote osteogenic differentiation and enhance ATF4 protein expression in vitro. a) ALP staining showing ALP activity in MC3T3‐E1 cells incubated with the small molecules OB‐1, OB‐2, OB‐3, and OB‐4 at 1 µm or DMSO for 48 h, respectively. b) Real‐time PCR analysis of osteocalcin mRNA level in MC3T3‐E1 cells after treatment with the small molecules OB‐1, OB‐2, OB‐3, and OB‐4 at 1 µm or vehicle for 48 h, respectively. c) Representative Western blot (left) and quantification (right) of ATF4 protein level in MC3T3‐E1 cells after treatment with the small molecules OB‐1, OB‐2, OB‐3, and OB‐4 at 1 µm or vehicle for 48 h, respectively. d) Real‐time PCR analysis of ATF4 mRNA level in MC3T3‐E1 cells after treatment with small molecules OB‐1, OB‐2, OB‐3, and OB‐4 at 1 µm or vehicle for 48 h, respectively. e) ALP staining showing ALP activity in MC3T3‐E1 cells incubated with the small molecule OB‐4 at a series of concentrations (40, 60, 80 nm) or DMSO for 48 h. f) Real‐time PCR analysis of osteocalcin mRNA level in MC3T3‐E1 cells after treatment with the small molecule OB‐4 at a series of concentrations (40, 60, 80 nm) or vehicle for 48 h. g) Representative Western blot (left) and quantification (right) of ATF4 protein level in MC3T3‐E1 cells after treatment with the small molecule OB‐4 at a series of concentrations (40, 60, 80 nm) or vehicle for 48 h. h) Real‐time PCR analysis of ATF4 mRNA level in MC3T3‐E1 cells after treatment with the small molecule OB‐4 at a series of concentrations (40, 60, 80 nm) or DMSO for 48 h, respectively. The data are presented as the mean ± standard deviation. *p < 0.05, **p < 0.01, ***p < 0.001 versus vehicle group.

**Figure 6:**
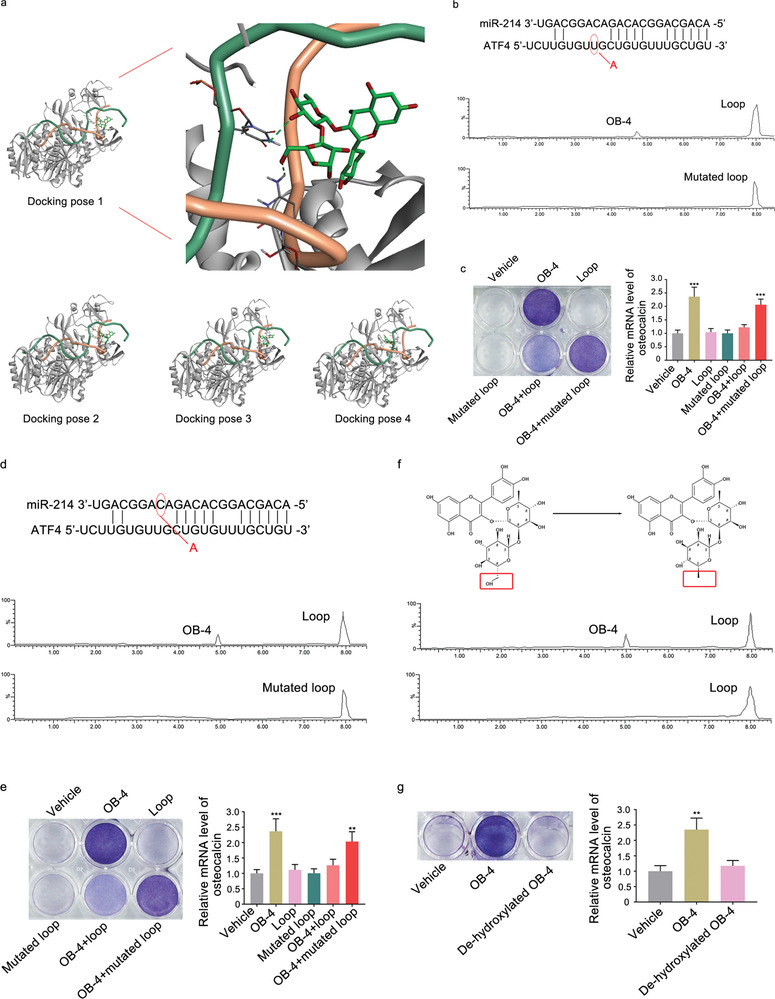
The mode of action for the optimal small molecule OB‐4. a) Left: Docking pose 1 shows OB‐4 (oxygen atoms in red, hydrogen atoms in white, carbon atoms in green) bound to the loop formed by miR‐214 (backbone: green ribbons) and ATF4 mRNA (backbone: orange ribbons) in the complex of AGO protein (grey ribbons). Docking pose 1 has the highest score among the four obtained docking poses. Right: A partial magnification of the docking pose 1 illustrates the binding sites between the small molecule OB‐4 and loop formed by miR‐214 and ATF4. Potential hydrogen‐bonding interactions between the small molecule OB‐4 and C15 and U9 of the miR‐214 and ATF4 loop are highlighted (green dashed lines). The other three computational docking poses show OB‐4 bound to the loop formed by miR‐214 and ATF4 mRNA in the complex of AGO protein. b) Pull‐down assay using streptavidin magnetic bead to examine the interaction between biotin‐labeled loop (formed by miR‐214 and mutated ATF4 mRNA) and the small molecule OB‐4. Small molecule OB‐4 captured by the mutated loop was examined by LC‐MS. c) ALP staining (left) and real‐time PCR analysis of osteocalcin mRNA level (right) in MC3T3‐E1 cells after treatment with vehicle, small molecule OB‐4, Loop, C15 mutated Loop, OB‐4 + Loop, and OB‐4 + C15 mutated loop, respectively. d) Pull‐down assay using streptavidin magnetic bead to examine the interaction between biotin‐labeled loop (formed by mutated miR‐214 and ATF4 mRNA) and the small molecule OB‐4. Small molecule OB‐4 captured by the mutated loop was examined by LC‐MS. e) ALP staining (left) and real‐time PCR analysis of osteocalcin mRNA level in MC3T3‐E1 cells after treatment with vehicle, small molecule OB‐4, Loop, U9 mutated Loop, OB‐4 + Loop, and OB‐4 + U9 mutated loop, respectively. f) Pull‐down assay using streptavidin to examine the interaction between biotin‐miR‐214‐ATF4 loop and the de‐hydroxylated small molecule OB‐4. De‐hydroxylated small molecule OB‐4 captured by the loop was examined by LC‐MS. g) ALP staining (left) and real‐time PCR analysis of osteocalcin mRNA level in MC3T3‐E1 cells after treatment with vehicle, small molecule OB‐4, and de‐hydroxylated OB‐4, respectively. *p < 0.05, **p < 0.01, ***p < 0.001 versus vehicle group.

The nontoxic concentration of the small molecules OB‐1, OB‐2, OB‐3, and OB‐4 was determined by cell proliferation (MTT) assay, respectively. The non‐toxic concentration 1 µm was adopted for further experiments (Figure S6, Supporting Information). After treatment of the small molecules OB‐1, OB‐2, OB‐3, and OB‐4, respectively, both alkaline phosphatase (ALP) activity and the level of osteocalcin mRNA in MC3T3‐E1 cells were measured. Compared to the corresponding control group, only the small molecule OB‐4 upregulated both the number of ALP staining positive cells (**Figure** **5**a) and the level of osteocalcin mRNA in MC3T3‐E1 cells (Figure 5b). After treatment of the small molecules OB‐1, OB‐2, OB‐3, and OB‐4, respectively, the amount of ATF4 protein and ATF4 mRNA level was measured. Compared to the corresponding control group, only the small molecule OB‐4 upregulated the amount of ATF4 protein (Figure 5c), but no change was found in the ATF4 mRNA level (Figure 5d). Furthermore, the dose‐effect pattern of the small molecule OB‐4 was investigated. Compared to the corresponding control group, the small molecule OB‐4 upregulated both the number of ALP staining positive cells (Figure 5e) and the level of osteocalcin mRNA in MC3T3‐E1 cells in a dose‐dependent manner, where the starting effective concentration for OB‐4 was 40 nm (Figure 5f). Consistently, the amount of ATF4 protein was upregulated (Figure 5g), but no change was found in the ATF4 mRNA level in a dose‐dependent manner, after treatment with the small molecule OB‐4 (Figure 5h). Next, docking method was used to predict the interaction between OB‐4 and miR‐214‐ATF4. In one of the four poses, it was noted that the hydroxy group of the small molecule OB‐4 could form hydrogen bonds with C15 and U9 base of the loop formed by miR‐214 and ATF4 mRNA (**Figure** **6**a). The biotin‐labeled miR‐214‐ATF4 loop with mutated either C15 or U9 was synthesized in vitro and incubated with the small molecule OB‐4. After that, streptavidin beads were added to the mixtures. LC‐MS was used to determine the binding ability. The LC‐MS data indicated that the mutation of C15 (Figure 6b) and U9 (Figure 6d) could abolish the binding between the small molecule OB‐4 and the loop, respectively. Compared to the small molecule OB‐4 treatment group, wild‐type biotin‐miR‐214‐ATF4 loop transfection group downregulated both the number of ALP staining positive cells and the level of osteocalcin mRNA in MC3T3‐E1 cells, whereas no significant change was found in either C15 or U9 mutated biotin‐miR‐214‐ATF4 loop transfection group (Figure 6c,e). The LC‐MS data revealed that de‐hydroxylated small molecule OB‐4 could not bind to miR‐214‐ATF4 loop in vitro (Figure 6f). Compared to the small molecule OB‐4 treatment group, the ability of the de‐hydroxylated small molecule OB‐4 in upregulating both the number of ALP staining positive cells and the level of osteocalcin mRNA in MC3T3‐E1 cells was attenuated, respectively (Figure 6g). Osteocalcin (Ocn) is a downstream molecule of ATF4 during osteoblast differentiation.^[^ ^13^ ^]^ It was further demonstrated that OB‐4‐induced ATF4 activation could up‐regulate Ocn expression in osteoblast in vitro (Figure S7, Supporting Information).

**Figure 7:**
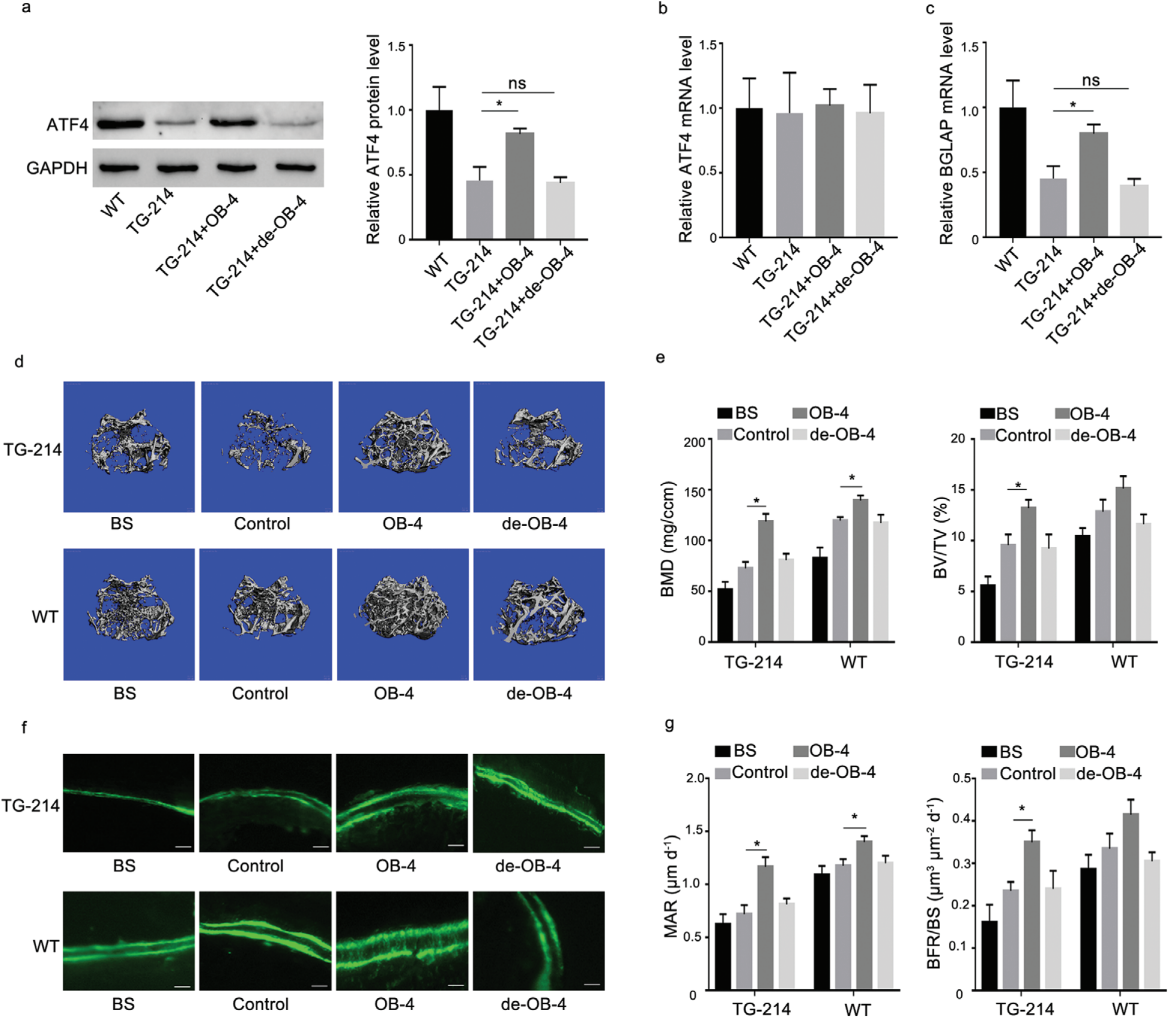
The effects of the small molecule OB‐4 on osteoblastic bone formation in osteoblast‐specific miR‐214 transgenic mice. a) Bone tissues levels of ATF4 protein were determined by Western blot analysis in both wild type mice (WT) and TG‐214 mice after administration of the (AspSerSer)_6_‐liposome (TG‐214), (AspSerSer)_6_‐liposome plus OB‐4 (TG‐214+OB‐4), and (AspSerSer)_6_‐liposome plus de‐hydroxylated OB‐4 (TG‐214+de‐OB‐4), respectively. b) Bone tissues levels of ATF4 mRNA were determined by qRT‐PCR in both wild type mice (WT) and TG‐214 mice after administration of the (AspSerSer)_6_‐liposome (TG‐214), (AspSerSer)_6_‐liposome plus OB‐4 (TG‐214+OB‐4), and (AspSerSer)_6_‐liposome plus de‐hydroxylated OB‐4 (TG‐214+de‐OB‐4), respectively. c) Bone tissues levels of BGLAP mRNA were determined by qRT‐PCR in both wild type mice (WT) and TG‐214 mice after administration of the (AspSerSer)_6_‐liposome (TG‐214), (AspSerSer)_6_‐liposome plus OB‐4 (TG‐214+OB‐4), and (AspSerSer)_6_‐liposome plus de‐hydroxylated OB‐4 (TG‐214+de‐OB‐4), respectively. d) Representative images showing the 3D trabecular architecture by micro‐CT reconstruction at the distal femur in both wild type mice (WT) and TG‐214 mice after administration of the (AspSerSer)_6_‐liposome (TG‐214), (AspSerSer)_6_‐liposome plus OB‐4 (TG‐214+OB‐4), and (AspSerSer)_6_‐liposome plus de‐hydroxylated OB‐4 (TG‐214+de‐OB‐4), respectively. Scale bars, 500 µm. e) microCT measurements for BMD and BV/TV at the distal femur in both wild type mice (WT) and TG‐214 mice after administration of the (AspSerSer)_6_‐liposome (TG‐214), (AspSerSer)_6_‐liposome plus OB‐4 (TG‐214+OB‐4), and (AspSerSer)_6_‐liposome plus de‐hydroxylated OB‐4 (TG‐214+de‐OB‐4), respectively. f) Representative fluorescent micrographs depicting new bone formation assessed by double calcein labeling. Scale bar, 20 µm. g) Dynamic bone histomorphometric measurements (MAR, BFR/BS) in both wild type mice (WT) and TG‐214 mice after administration of the (AspSerSer)_6_‐liposome (TG‐214), (AspSerSer)_6_‐liposome plus OB‐4 (TG‐214+OB‐4), and (AspSerSer)_6_‐liposome plus de‐hydroxylated OB‐4 (TG‐214+de‐OB‐4), respectively; *n* = 6 for each group. Data are means ± SD. *p < 0.05, ns denotes non‐significance (*p* > 0.05). Control: mice treated with (AspSerSer)_6_‐liposome as negative control.

To determine whether the small molecule OB‐4 could promote osteoblastic bone formation of TG‐214 mice, an osteoblast targeted delivery system (dioleoyl trimethylammonium propane‐based cationic liposomes attached to six repetitive sequences of aspartate, serine, serine (AspSerSer)_6_), was employed for encapsulating small molecule OB‐4, de‐hydroxylated OB‐4, respectively, specific to bone‐formation surfaces, as previously described.^[^ ^14^ ^]^ The WT and TG‐214 mice at 4 weeks old received tail intravenous injection of the (AspSerSer)_6_‐liposome plus small molecule OB‐4, (AspSerSer)_6_‐liposome plus de‐hydroxylated OB‐4 and (AspSerSer)_6_‐liposome, respectively, twice 1 week for 4 weeks, at a dose of 10 mg kg^−1^ for OB‐4 and de‐hydroxylated OB‐4, respectively. The intraosseous levels of ATF4 protein were upregulated in the TG‐214 mice treated with (AspSerSer)_6_‐liposome plus OB‐4, compare to that in the TG‐214 mice treated with (AspSerSer)_6_‐liposome, but not (AspSerSer)_6_‐liposome plus de‐hydroxylated OB‐4 (**Figure** **7**a). No significant differences in intraosseous levels of ATF4 mRNA were found among all the groups (Figure 7b). The real‐time PCR data demonstrated that the mRNA levels of BGLAP in bone tissues were significantly higher in the TG‐214 mice treated with (AspSerSer)_6_‐liposome plus OB‐4 than that in the TG‐214 mice treated with (AspSerSer)_6_‐liposome, but not in the TG‐214 mice treated with (AspSerSer)_6_‐liposome plus de‐hydroxylated OB‐4 (Figure 7c). The microCT data showed that both bone mineral density (BMD) and the ratio of bone volume to tissue volume (BV/TV) were significantly higher in the TG‐214 mice treated with (AspSerSer)_6_‐liposome plus OB‐4 than those in the TG‐214 mice treated with (AspSerSer)_6_‐liposome, but not in the TG‐214 mice treated with (AspSerSer)_6_‐liposome plus de‐hydroxylated OB‐4 (Figure 7d,e). The dynamic bone histomorphometric data showed that the bone formation parameters (mineral apposition rate [MAR] and bone formation rate [BFR]/BS) were significantly higher in the TG‐214 mice treated with (AspSerSer)_6_‐liposome plus OB‐4 than those in the TG‐214 mice treated with (AspSerSer)_6_‐liposome, but not in the TG‐214 mice treated with (AspSerSer)_6_‐liposome plus de‐hydroxylated OB‐4 (Figure 7f,g).

### 2.4 Determination of Small Molecule OC‐3 That Targets miR‐214‐TRAF3 Loop for Rescuing Bone Phenotype in Osteoclast‐Specific miR‐214 Knock in Mice

**Figure 8:**
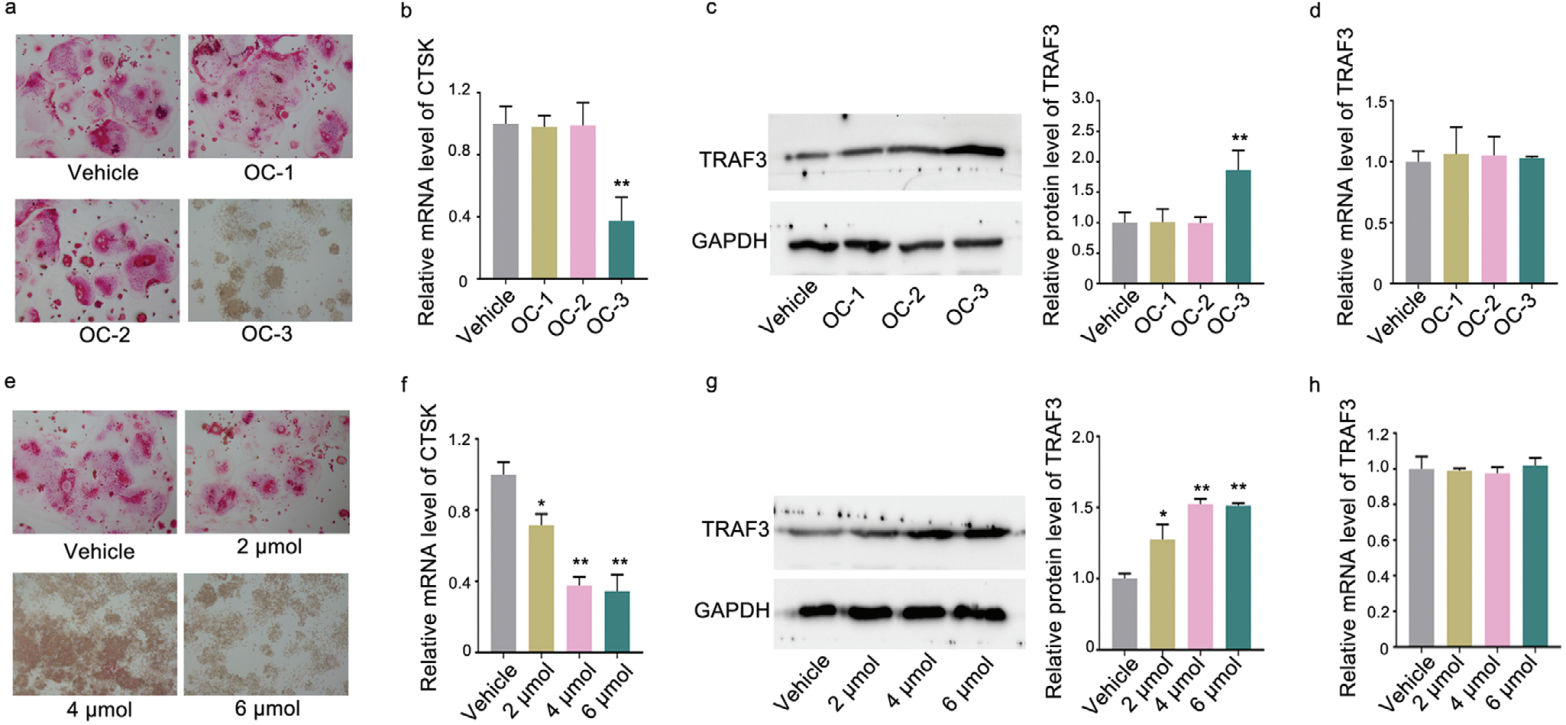
Small molecule OC‐3 could inhibit osteoclast activity and promote TRAF3 protein expression in vitro. a) TRAP staining in RAW 264.7 cells incubated with the small molecules OC‐1, OC‐2, and OC‐3 at 10 µm or vehicle for 7 days. b) Real‐time PCR analysis of CTSK mRNA level in RAW 264.7 cells after treatment with the small molecules OC‐1, OC‐2, OC‐3 at 10 µm or vehicle for 48 h. c) Representative western blot (left) and quantification (right) of TRAF3 protein level in RAW 264.7 cells after treatment with the small molecules OC‐1, OC‐2, and OC‐3 at 10 µm or vehicle for 48 h. d) Real‐time PCR analysis of TRAF3 mRNA level in RAW 264.7 cells after treatment with the small molecules OC‐1, OC‐2, OC‐3 at 10 µm or vehicle for 48 h, respectively. e) TRAP staining (left) in RAW 264.7 cells incubated with the small molecule OC‐3 at a series of concentrations (2, 4, 6 µm) or vehicle for 7 days. f) Real‐time PCR analysis of CTSK mRNA level in RAW 264.7 cells after treatment with the small molecule OC‐3 at a series of concentrations (2, 4, 6 µm) or vehicle for 48 h. g) Representative western blot (left) and quantification (right) of TRAF3 protein level in RAW 264.7 cells after treatment with the small molecule OC‐3 at a series of concentrations (2, 4, 6 µm) or vehicle for 48 h. h) Real‐time PCR analysis of TRAF3 mRNA level in RAW 264.7 cells after treatment with the small molecule OC‐3 at a series of concentrations (2, 4, 6 µm) or vehicle for 48 h, respectively. The data are presented as the mean ± standard deviation. *p < 0.05, **p < 0.01 versus vehicle group.

**Figure 9:**
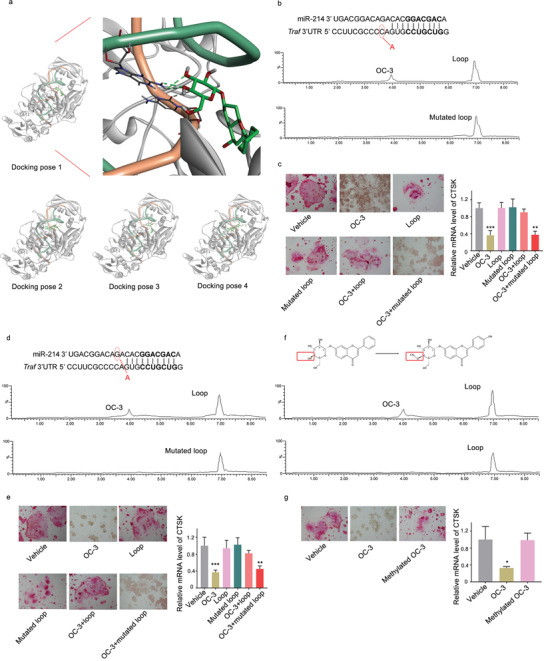
The mode of action for the optimal small molecule OC‐3. a) Left: Docking pose 1 shows OC‐3 (oxygen atoms in red, hydrogen atoms in white, carbon atoms in green) bound to the loop formed by miR‐214 (backbone: green ribbons) and TRAF3 mRNA (backbone: orange ribbons) in the complex of AGO protein (grey ribbons). Docking pose 1 has the highest score among the four obtained docking poses. Right: A partial magnification of the docking pose 1 illustrates the binding sites between the small molecule OC‐3 and loop formed by miR‐214 and TRAF3. Potential hydrogen‐bonding interactions between the small molecule OC‐3 and G13 and C10 base of the loop are highlighted (green dashed lines). The other three computational docking poses showing OC‐3 bound to the loop formed by miR‐214 and TRAF3 mRNA in the complex of AGO protein. b) Pull‐down assay using streptavidin magnetic bead to examine the interaction between biotin‐labeled loop (formed by miR‐214 and mutated TRAF3 mRNA) and the small molecule OC‐3. Small molecule OC‐3 captured by the mutated loop was examined by LC‐MS. c) TRAP staining (left) and real‐time PCR analysis of CTSK mRNA level (right) in RAW 264.7 cells after treatment with vehicle, small molecule OC‐3, Loop, G13 mutated loop, OC‐3 + loop, and OC‐3 + G13 mutated loop, respectively. d) Pull‐down assay using streptavidin magnetic bead to examine the interaction between biotin‐labeled loop (formed by mutated miR‐214 and TRAF3 mRNA) and the small molecule OC‐3. Small molecule OC‐3 captured by the mutated loop was examined by LC‐MS. e) TRAP staining (left) and real‐time PCR analysis of CTSK mRNA level in RAW 264.7 cells after treatment with vehicle, small molecule OC‐3, Loop, C10 mutated loop, OC‐3 + loop, and OC‐3 + C10 mutated loop, respectively. f) Pull‐down assay using streptavidin to examine the interaction between biotin‐miR‐214‐TRAF3 loop and the methylated small molecule OC‐3. Methylated small molecule OC‐3 captured by the loop was examined by LC‐MS. g) TRAP staining (left) and real‐time PCR analysis of CTSK mRNA level in RAW 264.7 cells after treatment with vehicle, small molecule OC‐3, and methylated small molecule OC‐3, respectively. *p < 0.05, **p < 0.01, ***p < 0.001 versus vehicle group.

The non‐toxic concentration of the small molecules OC‐1, OC‐2, OC‐3 was determined by MTT assay. The non‐toxic concentration of 10 µm was adopted for further experiments (Figure S8, Supporting Information). After treatment of the small molecules OC‐1, OC‐2, OC‐3, respectively, both TRAP staining and the level of CTSK mRNA in RAW 264.7 cells were measured. Compared to the corresponding control group, only the small molecule OC‐3 downregulated both the number of TRAP staining positive cells (**Figure** **8**a) and the level of CTSK mRNA in RAW 264.7 cells (Figure 8b). Compared to the corresponding control group, only the small molecule OC‐3 upregulated the amount of TRAF3 protein (Figure 8c), but no change was found in the TRAF3 mRNA level (Figure 8d). Furthermore, the dose‐effect pattern of the small molecule OC‐3 was investigated. Compared to the corresponding control group, the small molecule OC‐3 downregulated both the number of TRAP staining positive cells (Figure 8e) and the level of CTSK mRNA in RAW 264.7 cells in a dose‐dependent manner, where the starting effective concentration for OC‐3 was 2 µm (Figure 8f). Consistently, the amount of TRAF3 protein was upregulated (Figure 8g), but no change was found in the TRAF3 mRNA level in a dose‐dependent manner, after treatment with the small molecule OC‐3 (Figure 8h). Next, docking method was used to predict the interaction between OC‐3 and miR‐214‐TRAF3. This method yielded four poses with high docking scores for how OC‐3 and miR‐214‐TRAF3 may interact (**Figure** **9**a). In one of the four poses, it was noted that the hydroxy group of the small molecule OC‐3 could form hydrogen bonds with G13 and C10 base of the loop formed by miR‐214 and TRAF3 mRNA (Figure 9a). The biotin‐labeled miR‐214‐TRAF3 loop with mutated either G13 or C10 was synthesized in vitro and incubated with the small molecule OC‐3. After that, streptavidin beads were added to the mixtures. LC‐MS was used to determine the binding ability. The LC‐MS data indicated that the mutation of G13 (Figure 9b) and C10 (Figure 9d) could abolish the binding between the small molecule OC‐3 and the loop, respectively. Compared to the small molecule OC‐3 treatment group, wild‐type miR‐214‐TRAF3 loop transfection group upregulated both the number of TRAP staining positive cells and the level of CTSK mRNA in RAW 264.7 cells, whereas no significant change was found in either G13 or C10 mutated miR‐214‐TRAF3 loop transfection group (Figure 9c,e). The LC‐MS data revealed that methylated small molecule OC‐3 could not bind to miR‐214‐TRAF3 loop in vitro (Figure 9f). Compared to the small molecule OC‐3 treatment group, the ability of the methylated small molecule OC‐3 in downregulating both the number of TRAP staining positive cells and the level of CTSK mRNA in RAW 264.7 cells was attenuated, respectively (Figure 9g). Nuclear factor‐kappa B (NF‐*κ*B) is the downstream signaling pathway of TRAF3.^[^ ^11b^ ^]^ OC‐3‐induced TRAF3 upregulation could significantly inhibit the signaling pathway of NF‐*κ*B to downregulate the expression of the downstream factor p65 in osteoclast in vitro (Figure S9, Supporting Information).

**Figure 10:**
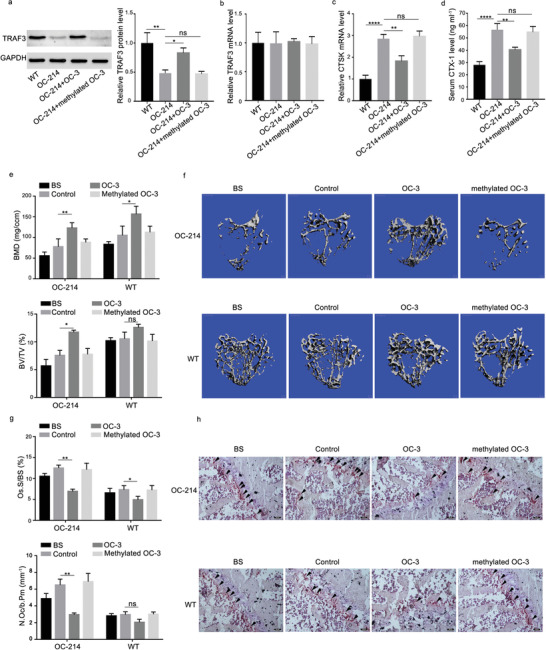
The effects of the small molecule OC‐3 on osteoclastic bone resorption in osteoclast‐specific miR‐214 knock‐in mice. a) Bone tissues levels of TRAF3 protein were determined by western blot analysis in both wild type mice (WT) and OC‐214 mice after administration of the (D‐Asp_8_)‐liposome (OC‐214), (D‐Asp_8_)‐liposome plus OC‐3 (OC‐214+OC‐3), and (D‐Asp_8_)‐liposome plus methylated OC‐3 (OC‐214+methylated OC‐3), respectively. b) Bone tissues levels of TRAF3 mRNA were determined by qRT‐PCR in both WT mice and OC‐214 mice after administration of the (D‐Asp_8_)‐liposome (OC‐214), (D‐Asp_8_)‐liposome plus OC‐3 (OC‐214+OC‐3), and (D‐Asp_8_)‐liposome plus methylated OC‐3 (OC‐214+methylated OC‐3), respectively. c) Bone tissues levels of CTSK mRNA were determined by qRT‐PCR in both WT mice and OC‐214 mice after administration of the (D‐Asp_8_)‐liposome (OC‐214), (D‐Asp_8_)‐liposome plus OC‐3 (OC‐214+OC‐3), and (D‐Asp_8_)‐liposome plus methylated OC‐3 (OC‐214+methylated OC‐3), respectively. d) Serum levels of CTX‐1 were determined by ELISA in both WT mice and OC‐214 mice after administration of the (D‐Asp_8_)‐liposome (OC‐214), (D‐Asp_8_)‐liposome plus OC‐3 (OC‐214+OC‐3), and (D‐Asp_8_)‐liposome plus methylated OC‐3 (OC‐214+methylated OC‐3), respectively. e) microCT measurements for BMD and BV/TV in proximal tibia in both WT and OC‐214 mice after administration of the (D‐Asp_8_)‐liposome (OC‐214), (D‐Asp_8_)‐liposome plus OC‐3 (OC‐214+OC‐3), and (D‐Asp_8_)‐liposome plus methylated OC‐3 (OC‐214+methylated OC‐3), respectively. f) Representative images showing the 3D trabecular architecture by micro‐CT reconstruction in proximal tibia in both WT and OC‐214 mice after administration of the (D‐Asp_8_)‐liposome (OC‐214), (D‐Asp_8_)‐liposome plus OC‐3 (OC‐214+OC‐3), and (D‐Asp_8_)‐liposome plus methylated OC‐3 (OC‐214+methylated OC‐3), respectively. Scale bars, 500 µm. g) Bone histomorphometric measurements (Oc.S/BS and N.Oc/B.Pm) in proximal tibia in both WT and OC‐214 mice after administration of the (D‐Asp_8_)‐liposome (OC‐214), (D‐Asp_8_)‐liposome plus OC‐3 (OC‐214+OC‐3), and (D‐Asp_8_)‐liposome plus methylated OC‐3 (OC‐214+methylated OC‐3), respectively. h) The representative TRAP staining images for the trabecular bone in proximal tibia in both WT and OC‐214 mice after administration of the (D‐Asp_8_)‐liposome (OC‐214), (D‐Asp_8_)‐liposome plus OC‐3 (OC‐214+OC‐3), and (D‐Asp_8_)‐liposome plus methylated OC‐3 (OC‐214+methylated OC‐3), respectively. Scale bar: 100 µm. Arrow indicates TRAP+ cells. Note: *n* = 6 for each group. Data were means ± SD. *p < 0.05, **p < 0.01, ***p < 0.001, ****p < 0.0001, ns denotes non‐significance. BS: mice that were sacrificed before treatment as baseline; control: mice treated with (D‐Asp_8_)‐liposome as negative control.

To determine whether the small molecule OC‐3 could attenuate osteoclastic bone resorption in OC‐214 mice, an osteoclast targeted delivery system (D‐Asp_8_ peptide with liposome) was employed for encapsulating the small molecule OC‐3 and methylated OC‐3, respectively, specific to bone‐resorption surfaces, as previously described.^[^ ^15^ ^]^ The WT and OC‐214 mice at 4 weeks old received tail intravenous injection of the (D‐Asp_8_)‐liposome plus small molecule OC‐3, (D‐Asp_8_)‐liposome plus methylated OC‐3, and (D‐Asp_8_)‐liposome, respectively, twice 1 week for 4 weeks, at a dose of 20 mg kg^−1^ for OC‐3 and methylated OC‐3, respectively. The intraosseous levels of TRAF3 protein were upregulated in the OC‐214 mice treated with (D‐Asp_8_)‐liposome plus small molecule OC‐3, compared to that in the OC‐214 mice treated with (D‐Asp_8_)‐liposome, but not (D‐Asp_8_)‐liposome plus methylated OC‐3 (**Figure** **10**a). No significant differences in intraosseous levels of TRAF3 mRNA were found among all the groups (Figure 10b). The real‐time PCR data demonstrated that the mRNA levels of CTSK in bone tissues were significantly lower in the OC‐214 mice treated with (D‐Asp_8_)‐liposome plus small molecule OC‐3 than that in the OC‐214 mice treated with (D‐Asp_8_)‐liposome, but not in the OC‐214 mice treated with (D‐Asp_8_)‐liposome plus methylated OC‐3 (Figure 10c). The enzyme linked immunosorbent assay (ELISA) data demonstrated that the levels of CTX‐1 in serum were significantly lower in the OC‐214 mice treated with (D‐Asp_8_)‐liposome plus small molecule OC‐3 than that in the OC‐214 mice treated with (D‐Asp_8_)‐liposome, but not in the OC‐214 mice treated with (D‐Asp_8_)‐liposome plus methylated OC‐3 (Figure 10d). The microCT data showed that both BMD and BV/TV were significantly higher in the OC‐214 mice treated with (D‐Asp_8_)‐liposome plus small molecule OC‐3 than that in the OC‐214 mice treated with (D‐Asp_8_)‐liposome, but not in the OC‐214 mice treated with (D‐Asp_8_)‐liposome plus methylated OC‐3 (Figure 10e,f). The bone histomorphometric data showed that both Oc.S/BS and N.Oc/B.Pm were significantly lower in the OC‐214 mice treated with (D‐Asp_8_)‐liposome plus small molecule OC‐3 than that in the OC‐214 mice treated with (D‐Asp_8_)‐liposome, but not in the OC‐214 mice treated with (D‐Asp_8_)‐liposome plus methylated OC‐3 (Figure 10g). The TRAP staining data showed that the numbers of TRAP staining positive cells were significantly lower in the OC‐214 mice treated with (D‐Asp_8_)‐liposome plus small molecule OC‐3 than that in the OC‐214 mice treated with (D‐Asp_8_)‐liposome, but not in the OC‐214 mice treated with (D‐Asp_8_)‐liposome plus methylated OC‐3 (Figure 10h).

### 2.5 Determination of the Specificity of OB‐4 and OC‐3

**Figure 11:**
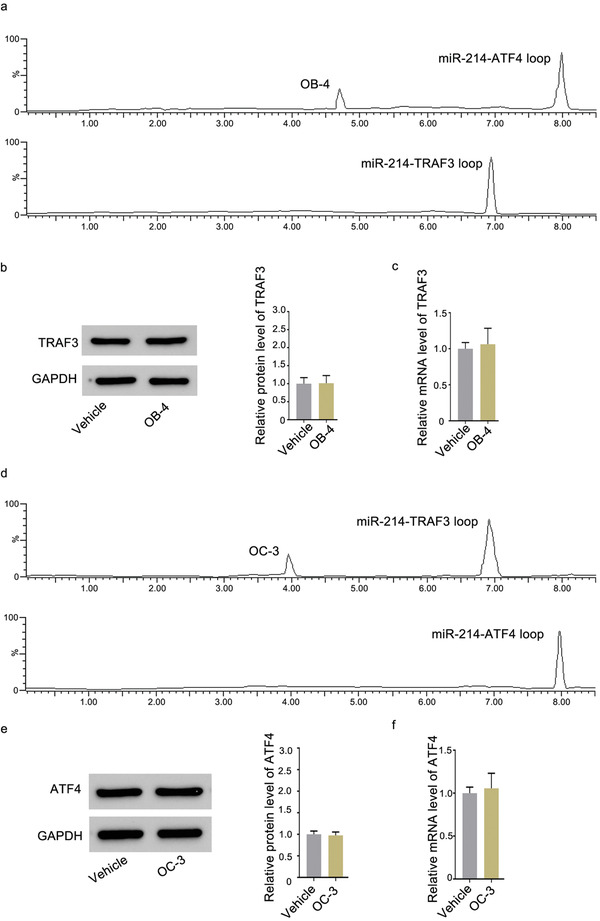
Determination of the specificity of OB‐4 and OC‐3. a) Pull‐down assay was performed using streptavidin magnetic bead to examine the interaction between biotin‐miR‐214‐TRAF3 loop and small molecule OB‐4. Small molecule OB‐4 captured by biotin‐miR‐214‐TRAF3 loop was examined by LC‐MS. b) Representative western blot (left) and quantification (right) of TRAF3 protein level in MC3T3‐E1 cells after treatment with the small molecule OB‐4 at 80 nm or vehicle for 48 h. c) Real‐time PCR analysis of TRAF3 mRNA level in MC3T3‐E1 cells after treatment with the small molecule OB‐4 at 80 nm or vehicle for 48 h. d) Pull‐down assay was performed using streptavidin magnetic bead to examine the interaction between biotin‐miR‐214‐ATF4 loop and small molecule OC‐3. Small molecule OC‐3 captured by biotin‐miR‐214‐ATF4 loop was examined by LC‐MS. e) Representative western blot (left) and quantification (right) of ATF4 protein level in RAW 264.7 cells after treatment with the small molecule OC‐3 at 6 µm or vehicle for 48 h. f) Real‐time PCR analysis of ATF4 mRNA level in RAW 264.7 cells after treatment with small molecule OC‐3 at 6 µm or vehicle for 48 h, respectively.

To further determine the specificity of the targeting, the effect of OB‐4 on TRAF3 protein expression was explored. The small molecule OB‐4 and OC‐3 could not bind to miR‐214‐TRAF3 loop and miR‐214‐ATF4 loop in vitro, as evidenced by the LC‐MS data (**Figure** **11**a,b). After treatment of the small molecule OB‐4 and OC‐3, the levels of TRAF3 and ATF4 protein and TRAF3 and ATF4 mRNA in MC3T3‐E1 cells and RAW 264.7 cells were measured. Compared to the corresponding control group, the small molecule OB‐4 and OC‐3 could not upregulate both the level of TRAF3 protein and ATF4 protein (Figure 11b,d) and the TRAF3 and ATF4 mRNA (Figure 11c,f), respectively, in MC3T3‐E1 cells and RAW 264.7 cells.

To further determine the specificity of the targeting, the effect of OB‐4 on protein expression of other target mRNAs of miR‐214 was explored. Previously published studies have demonstrated that miR‐214 could target Osterix,^[^ ^16^ ^]^ PTEN,^[^ ^17^ ^]^ FGFR1,^[^ ^18^ ^]^ *β*‐catenin,^[^ ^19^ ^]^ PDRG1,^[^ ^20^ ^]^ FOXM1,^[^ ^21^ ^]^ CDC25B,^[^ ^22^ ^]^ CUG‐BP1,^[^ ^23^ ^]^ respectively. The small molecule OB‐4 and OC‐3 could not bind to the loops formed by miR‐214 and its other target mRNAs, including Osterix, PTEN, FGFR1, *β*‐catenin, PDRG, FOXM1, CDC25B, CUG‐BP1, in vitro, as evidenced by the LC‐MS data (Figures S10 and S11, Supporting Information). After treatment of the small molecule OB‐4 and OC‐3, respectively, both the mRNA levels and protein levels of Osterix, PTEN, FGFR1, *β*‐catenin, PDRG, FOXM1, CDC25B, CUG‐BP1 in MC3T3‐E1 cells, and in RAW 264.7 were measured, respectively. Compared to the corresponding control group, the small molecule OB‐4 and OC‐3 could not regulate both the mRNA levels and protein levels in MC3T3‐E1 cells and in RAW 264.7 cells, respectively (Figures S10 and S11, Supporting Information).

To validate the specific binding of the small molecule OB4 and the small molecule OC‐3 with the miR‐214‐ATF4 and miR‐214‐TRAF3 mRNA complex, respectively, both SPR assay and isothermal titration calorimetry (ITC) assays were performed. The SPR data showed a high affinity interaction between the small molecule OB‐4 and miR‐214‐ATF4 mRNA of 2.13 ± 0.35 µm, OC‐3 and miR‐214‐TRAF3 mRNA of 18.6 ± 6.4 µm (Figure S12a, Supporting Information). The ITC assay indicated a binding for OB‐4 targeting miR‐214‐ATF4 mRNA and OC‐3 targeting miR‐214‐TRAF3 mRNA, respectively (Figure S12b, Supporting Information). As evidenced by the data from both SPR assay and ITC assay, the small molecule OB‐4 and the small molecule OC‐3 could specifically bind with miR‐214‐ATF4 mRNA and miR‐214‐TRAF3 mRNA, respectively.

## 3 Discussion

This study is for the first time to develop a loop‐based and AGO‐incorporated virtual model for screening small‐molecule candidates targeting the loop motifs in AGO‐mediated miRNA–mRNA interaction to block translation repression of a specific mRNA. The virtual screening model was a combination of knowledge‐based and structure‐based models. The knowledge‐based model provided the possible miRNA–mRNA–small molecules interactions with query features, whereas the structure‐based model produced the possible complex with different binding energy scores. The intersection data of both models provided the reliable candidates. The strategy was successfully applied in this study to virtually screen the potential small molecules targeting miRNA–mRNA interactions.

Currently, there are three prediction methods for RNA secondary structure, each with its own characteristics. RNAfold predicted the minimum free energy structure using the thermodynamic parameter approach.^[^ ^24^ ^]^ Mfold was based on dynamic programming methods.^[^ ^25^ ^]^ AveRNA a comprehensive web method combined 11 other prediction programs to predict secondary structures.^[^ ^26^ ^]^ There was no source code of AveRNA, whereas source code was available for RNAfold. Considering that the prediction data of RNAfold was almost the same with AveRNA and Mfold (Table S3, Supporting Information) and RNAfold source code was easy to install to cooperate with the local machine learning algorithms scripts, RNAfold was employed in our study to provide the secondary structure of miRNA–mRNA single sequence. In general, miRNA and mRNA were adopted to predict their own structure in a single sequence as the input. Here, we first combined mRNA and miRNA sequence with random number of placeholders so that the mRNA‐placeholders‐miRNA sequence interaction formed, treated as the input format. The single miRNA–mRNA interaction contained no placeholder, while the miRNA‐placeholders‐mRNA contained several placeholders. Such strategy was adopted that miRNA–mRNA interaction sequence treated as the input of secondary structure prediction programs, which was supported by the research strategy of the miRNA target identification,^[^ ^5^ ^]^ that the placeholders were replaced with ligation site and the secondary structure (formed loops) appeared.

In this loop‐based and AGO‐incorporated virtual model, we adopted the loop formed by mature miRNA and their specific target mRNA as a postulated target. First, the loop could be a structurally unique target, as evidenced by the uniqueness of the position, size, shape, and base composition of the loop. Second, the loop is functionally important, as evidenced by the elevated expression levels of the corresponding proteins translated from the target mRNAs after point mutations on the loops in ten most widely reported miRNA–mRNA interactions,^[^ ^27^ ^]^ without altered expression levels of the target mRNAs. Third, the loop is highly stable, as evidenced by the low energy of the miRNA–mRNA interactions by calculation. Fourth, the loop is highly specific, as evidenced by the two different small molecule candidates (OB‐4 and OC‐3) targeting the same miRNA (miR‐214) with different target mRNA (ATF4 and TRAF3) in the loop, respectively. Being the same miRNA, the role of miR‐214 is distinctive in different cells where its main target genes are different. Blocking the maturation of one miRNA will no doubt causes massive changes in downstream target genes. Herein, the unique small molecule obtained from our model that targets miR‐214‐ATF4 and miR‐214‐TRAF3, respectively, indicates the specificity of the screening strategy. According to the calculation of the variable importance measures,^[^ ^28^ ^]^ the miRNA–mRNA features whose measures above 0.001 explained the specificity of loops sufficiently. Therefore, it is feasible to take the loop formed by mature miRNA and their specific target mRNA as a target for screening small molecules.

To validate the utility of our loop‐based and AGO‐incorporated virtual screening model, the interaction of miR‐214‐ATF4 was adopted as a target in osteoblast‐like cell. After the extensive search of the database, the top ten small molecule candidates with high possibility that bind to miR‐214‐ATF4 were predicted. Experimentally, the small molecules OB‐1, OB‐2, OB‐3, and OB‐4 could bind to miR‐214‐ATF4, respectively, as indicated by the LC‐MS. The further functional data demonstrated that only small molecule OB‐4 treatment could enhance ALP staining and elevate level of osteocalcin mRNA in MC3T3‐E1 cells. In addition, treatment with small molecule OB‐4 upregulated the amount of ATF4 protein, but no change was found in the ATF4 mRNA level. These data suggested that the predicted small molecule OB‐4 could target miR‐214‐ATF4 and then release the translation repression of ATF4 mRNA. To prove the specificity, the mutated miR‐214‐ATF4 loop was synthesized. It was found that small molecule OB‐4 could not bind to the mutated loop. Moreover, the de‐hydroxylated small molecule OB‐4 could not bind to the miR‐214‐ATF4 loop. Furthermore, the effect of OB‐4 on both the mRNA levels and the corresponding protein levels of other target mRNAs of miR‐214 was explored. The unchanged levels of both the mRNA levels and the corresponding protein levels of other target mRNAs of miR‐214 after OB‐4 treatment indicates the specificity of the targeting. Furthermore, the data from both SPR assay and ITC assay consistently indicated that the small molecule OB‐4 could specifically bind with miR‐214‐ATF4 mRNA.

To further confirm the utility of the model, the interaction of miR‐214‐TRAF3 was adopted as a target in osteoclast‐like cell. After the extensive search of the database, the top ten small molecule candidates with high possibility that bind to miR‐214‐TRAF3 were predicted. Experimentally, the small molecules OC‐1, OC‐2, and OC‐3 could bind to miR‐214‐TRAF3, respectively, as indicated by the LC‐MS. The further functional data demonstrated that only small molecule OC‐3 treatment could downregulate both the number of TRAP staining positive cells and the level of CTSK mRNA in RAW 264.7 cells. In addition, treatment with small molecule OC‐3 upregulated the amount of TRAF3 protein, but no change was found in the TRAF3 mRNA level. These data suggested that the predicted small molecule OC‐3 could target miR‐214‐TRAF3 and then release the translation repression of TRAF3 mRNA. To prove the specificity, the mutated miR‐214‐TRAF3 loop was synthesized. It was found that the small molecule OC‐3 could not bind to the mutated loop. Moreover, the methylated small molecule OC‐3 could not bind to the miR‐214‐TRAF3 loop. Furthermore, the effect of OC‐3 on both the mRNA levels and the corresponding protein levels of other target mRNAs of miR‐214 was explored. The unchanged levels of both the mRNA levels and the corresponding protein levels of other target mRNAs of miR‐214 after OC‐3 treatment indicates the specificity of the targeting. Furthermore, the data from both SPR assay and ITC assay consistently indicated that the small molecule OC‐3 could specifically bind with miR‐214‐TRAF3 mRNA.

To detect the potential interaction among AGO2, miR‐214, and the small molecule OB‐4, AGO2–small molecule interaction assay was performed. Compared to the control MC3T3‐E1 cells, the higher amount of the small molecule OB‐4 attached to AGO2 in miR‐214‐overexpressing MC3T3‐E1 cells was found, indicating that the small molecule OB‐4 could be involved in the interaction among AGO2‐miR‐214‐ATF4 mRNA inside cells. Consistently, compared to the control RAW 264.7 cells, the higher amount of the small molecule OC‐3 attached to AGO2 in miR‐214‐overexpressing RAW 264.7 cells was found, indicating that the small molecule OC‐3 could be involved in the interaction among AGO2‐miR‐214‐TRAF3 mRNA inside cells. In re‐analyzing the model, it was found that only the small molecules OB‐4 and OC3 have high scores in both the knowledge‐based algorithm and the structure‐based algorithm, while the small molecules OB‐1, OB‐2, OB‐3, OC‐1, and OC‐2 only have high scores in the knowledge‐based algorithm or in the structure‐based algorithm. Thus, it was believed that incorporating the AGO protein in calculating the binding energy of AGO‐miRNA‐target mRNA small molecule complex in our algorithm could facilitate identifying the small molecule candidates targeting the proposed miRNA–mRNA interaction. Such data further demonstrated the necessity in adopting both the knowledge‐based algorithm and the structure‐based algorithm in our model.

In this study, loop formed by miRNA and mRNA was first identified as a specific target for screening small molecules targeting miRNA. The published 1936 RNA motif small molecule interactions developed by Disney et al. was employed in the knowledge‐based algorithm for our virtual screening model.^[^ ^2b,c^ ^]^ Moreover, structure‐based algorithm was also developed to integrate AGO protein to our virtual screening model to enhance the hit rate. Based on the available RNA–small molecule interaction data as the source, machine learning was applied to predict miRNA–mRNA–small molecule interactions. The assumption for the machine learning is that joined miRNA–mRNA sequences have similar small molecule binding prosperities with that of RNA. In order to obtain high quality prediction model, multiple measures, including adjustment of positive/negative ratio for the imbalanced data, multiple model comparison, and hyper‐parameter optimizations, were implemented. To screen small molecule databases, miRNA–mRNA sequences were joined from 5′ to 3′ and treat them as a single chain. The features of miRNA–mRNA and the features of small molecules were joined as input for the prediction.

The structure‐based algorithm was achieved by the application of AutoDock Vina to calculate the average binding energy values of each mode of small molecule candidates as the scores. Autodock Vina could also provide the potential binding sites (pockets, cavities, conserved amino acid residues, etc) and then dock the ligands in receptors. The knowledge‐based model could only provide the possible miRNA–mRNA–small molecules interactions with query features. To acquire the information of binding sites, it was necessary to employ the AutoDock Vina to provide the potential binding sites as the supplement and validation. Only the small molecules–miRNA–mRNA interactions supported by the potential binding sites could be reliable and explicable.

To be noted, miR‐214 affects mRNA expression of TRAF3 in two published papers,^[^ ^29^ ^]^ while here miR‐214 had no effect on TRAF3 mRNA level. Based on our published data^[^ ^10^ ^]^ and the data published by others,^[^ ^29^ ^]^ it suggested that the regulation mode of miRNA on its target genes could be cell‐type specific. In some type of cells, the transcription process of the target genes could not be affected, but the translation process is affected.^[^ ^10^ ^]^ In some type of cells, both the transcription and translation processes of the target genes are affected.^[^ ^29^ ^]^ So, the cell‐type specific regulation mode of miRNA on its target genes should be taken into consideration before virtual screening.

The region where miRNA is loaded into AGO has been found to be close to the region where miRNA (5′‐UTR of the miRNA) forms the loop with its target mRNA.^[^ ^3^, ^30^ ^]^ Two interaction mechanisms of action of the predicted small molecules that block the miRNA repression to specific mRNA are proposed (Figure S13, Supporting Information). First, the predicted small molecule targeting the loop could interfere the loop formation of the miRNA with the target mRNA when the miRNA directed the AGO to the target mRNA. Second, this targeting could sterically interfere AGO nearby, therefore, the repression of mRNA translation by AGO could be rescued.

## 4 Conclusion

These data indicated that our loop‐based and AGO‐incorporated virtual screening model could help to obtain small‐molecule candidates specifically targeting AGO‐mediated miR‐214‐mRNA interaction to rescue bone phenotype in genetically modified mice.

## 5 Experimental Section

##### Definition of Loop

Those imperfect hybrids of the miRNA with the target mRNA sequences could form loop.^[^ ^3^, ^30^ ^]^ To identify the loop using the computation strategy, the sequence of the miRNA and its target mRNA was first input; then the unpaired bases (loops) were generated by the database (Figure S14, Supporting Information).

##### Calculation of Minimum Free Energy of RNA Sequences

Secondary structure on sequences is the list of canonical base pairs. Assuming an RNA sequence R = r_1_r_2_r_3_…r_n_, r_i_∈{A,G,C,U}, (i,j) is a base pair between r_i_ and r_j_ (i<j).There are few constraints:

1) There are at least three bases apart i‐j>3 in pairing bases
2) For any i, there exists at most one k ≠ i − l, i + l, makes base pair (i,k) with i
3) If (i,j) and(k,l)∈R, and i<k, either i<k<l<j or i<j<k<l

The first condition means sharp U‐turns are prohibited. The second condition implies that each nucleotide can take part in not more than one base pair; the last condition forbids knots and pseudoknots. The last restriction is necessary for dynamic programming algorithms. A base pair (k, l) is interior to the base pair (i, j), if i < k < l < j. It is immediately interior if there is no base pair p, q such that i < p < k < l < q < j. For each base pair (i, j), the corresponding loop is defined as consisting of (i, j) itself, the base pairs immediately interior to (i, j), and all unpaired regions connecting these base pairs. The energy of the secondary structure is assumed to be the sum of the energy contributions of all loops. It is noted that a stacked base pair constitutes a loop of zero size. As a consequence of the additivity of the energy contributions, the minimum free energy can be calculated recursively by dynamic programming.

##### Prediction of Target Sites

To search for canonical seed matches and restricted non‐canonical sites and to obtain predicted and mutant miRNA::target duplexes, a modified version of the miRanda algorithm was used, using a score cutoff (‐sc) of 120 and gap opening and gap extension (‐go ‐ge) of ‐9 and ‐4, respectively. The modified version excludes the first 5′ base and last two 3′ bases of the microRNA from the alignment and allows for only a single G:U or mismatch in the seed region (positions 2 to 7). The algorithm computes an optimal sequence complementarity alignment between the microRNA and mRNA using a weighted dynamic programming approach, where matches in the seed regions have higher position‐specific weights, resulting in alignments that strongly favor 5′ base‐pairing. 3′‐UTR sequences were downloaded from UCSC genome browser, with the longest UTR chosen from alternative isoforms. “Canonical target” sites are defined as sites that contain minimally a 6‐mer perfect match at positions 2 to 7 of the microRNA.

##### Calculation of the Profiles of miRNA–mRNA Loops in Different miRNA Families

An RNA transcript consisting of *n* nucleotides was encoded by S ∈ {*A*, *C*, *G*, *U*}^*n*^ from 5′ to 3′ end. For a given pair of miRNA S^*miRNA*^ and mRNA S^*mRNA*^, we defined $E^{hybrid}\left[\begin{array}{cc} i, & k\\ j, & l \end{array}\right]$ as the minimal energy of any interaction of the subsequences $S_{i}^{miRNA}..S_{k}^{miRNA}$ and $S_{l}^{mRNA}..S_{j}^{mRNA}$ under the additional condition that the subsequence ends form each base pair, that is, $\left(S_{i}^{miRNA},\;S_{j}^{mRNA}\right)$ and $\left(S_{k}^{miRNA},S_{l}^{mRNA}\right)$, which was known as Watson–Crick or G‐U base pairs. This energy term also included the RNA–RNA interaction initiation energy penalty as well as base pair position penalty. The combination profile penalty was defined as $ED^{miRNA}\;\left[i,k\right]=\;-RT.\log\left(P_{r}^{u}\left[i..k\right]\right),$ in which $P_{r}^{u}\left[i..k\right]$ denotes the unpaired probability for subsequence $S_{i}^{miRNA}..S_{k}^{miRNA}$, *R* denotes the gas constant, and *T* denotes the temperature of the system. ED^*mRNA*^ was defined analogously. Here, ED^*dangle*^ was defined as the energy of flank ends between the inner loops. Using the ED^*miRNA*^ and ED^*mRNA*^ values for the probabilities of pair of miRNA S^*miRNA*^ and mRNA S^*mRNA*^, the combined energy profile could be calculated by

(1) $$\begin{array}{c} \begin{array}{c} E{\left[\begin{array}{cc} i, & k\\ j, & l \end{array}\right]}^{v2}=E^{hybrid}\;\left[\begin{array}{cc} i, & k\\ j, & l \end{array}\right]+ED^{miRNA}\left[i,k\right]\\ \;\;\;\;\;+P_{r}^{u}\left[i-1|i..k\right].E^{dangle}\left[S_{i-1}^{miRNA}\right]\\ \;\;\;\;\;+P_{r}^{u}\left[k+1|i..k\right].E^{dangle}\left[S_{k+1}^{miRNA}\right]\\ \;\;\;\;\;+ED^{mRNA}\left[j,l\right]+P_{r}^{u}\left[j-1|j..l\right].E^{dangle}\\ \;\;\;\;\;\left[S_{j-1}^{mRNA}\right]+P_{r}^{u}\left[l+1|j..l\right].E^{dangle}\left[S_{l+1}^{mRNA}\right] \end{array} \end{array}$$

##### Modeling miRNA–mRNA Complex

The miRNA–mRNA interaction feature was calculated as a single RNA sequence from 5′ to 3′, as indicated by the result of RNA secondary structure with different placeholders inserted in the binding site of mRNA–miRNA sequence. The RNAfold and RNAComposer were employed to model miR‐214–mRNA interaction. The 1st step was to predict the secondary structure of miRNA–mRNA sequence with RNAfold. The 2nd step was to predict the 3D structure of miRNA–mRNA interaction with RNAComposer, which required RNA sequence and secondary structure as the input.^[^ ^31^ ^]^

##### Docking of AGO2 and miR‐214‐mRNA

In many biological processes, nucleic acid‐protein interactions play important roles. In this study, NPDock was used with default parameters to achieve the docking of AGO2 and miR‐214‐mRNA. The 3D structure of miR‐214‐mRNA was loaded as the ligand and the AGO2 was loaded as the receptor. Then decoys were generated by GRAMM program. After scored and ranked with statistical potentials, the best‐scored decoys were clustered and refined. Finally, the complex structure of AGO2‐miR‐214‐mRNA in PDB format was obtained.

##### Docking of AGO2‐miR‐214‐mRNA with Small Molecules

It is necessary to score the docked “poses” between ligand and receptor. The strength of interaction between receptor and ligand determines the scoring and can be expressed as the free energy of binding.

(2) $$\begin{array}{c} \Delta G_{\mathrm{bind}}=\Delta G_{\mathrm{complex}}-\left(\Delta G_{\mathrm{receptor}}+\Delta G_{\mathrm{ligand}}\right) \end{array}$$

The AutoDock Vina (referred to as Vina here) was used to dock AGO2‐miR‐214‐mRNA binding sites with small molecules. The steps of the process were as below:

1) The macromolecule structure of AGO2‐miR‐214‐mRNA was loaded with AutoDockTools as receptor, the charges and hydrogen atoms were added, and non‐polar hydrogen atoms were merged.
2) The small molecules were drawn using ChemDraw 10.0, and their structures were optimized with MM2 method as ligand.
3) Both macromolecule and ligand structures were converted to PDBQT format using AutoDock tools.
4) Default Vina parameters were used to conduct search with maximum nine conformations. Top confirmations with the lowest binding energies were visualized with BIOVIA Discovery Studio.

##### RNA Sequence Features

The sequences of miRNA–mRNA were joined as a single RNA sequence from 5′ to 3′. Two categories of features were created for each RNA sequence, global sequence information feature, and secondary structure feature. The sequences of miRNA–mRNA were joined as a single RNA sequence from 5′ to 3′. Global sequence information features were those properties based on sequences, which include RNA length, GC content, ratio of each nucleotides, ratio of each combination of two neighboring nucleotides, and so on. The RNA feature set had 226 dimensions. Secondary structure features were based on the secondary structure of RNA sequences, which include number of bulges, number of base pairs, average number of base pairs in different window sizes, ratio of mono‐ and di‐nucleotide structures, and so on. The secondary structures were predicted by RNAfold in ViennaRNA Package.^[^ ^32^ ^]^

##### Small Molecule Features

For each small molecule, molecule fingerprints were calculated by Python RDkit tools using fingerprint size (bits) 1024, which were proven to effectively characterize molecule structures.

##### RNA–Small Molecule Interaction Features

RNA–small molecule interaction features were the joined information of both RNA sequence features and small molecule structure features. The information from both RNA and small molecules were used to build models and conduct predictions. Therefore, the feature set of RNA–small molecule had 1250 dimensions in total.

##### RNA–Small Molecule Interaction Dataset

All data were manually collected from other published journals and patents and they were stored in a database (datafile_refined.csv, https://github.com/wanyy063700/SMTRS/). Interactions that consisted of either RNAs that lacked valid sequences or small molecules with lack of valid structures were removed and the remaining 738 valid interactions were used for further process. All RNA and their corresponding small molecule were paired and labeled as “targeted”; their features were built based on the above methods. For non‐targeted data set, we used all RNA and all small molecules that were not paired with them, and randomly selected a part of them as training set. The ratio between targeted and non‐targeted pairs was set as 1:3. Data were split randomly with 4/5 as training set and rest 1/5 as test set. During model selection, training dataset was split into ten pieces for cross validation.

##### Machine Learning Method Selected for Prediction

In the study, seven machine learning methods were used for model selection, including logistic regression (LR), linear discriminant analysis, K‐nearest neighbors, classification and regression trees, naive Bayes, support vector machine, and random forest. After ten cross validations, the mean and STD values of accuracy and ros_auc were calculated with Morgan and RDkit fingerprints of small molecules, respectively. The selected values were bonded (mean > 0.8, STD < 0.2). According to the comprehensive comparison of the bonded results, random forest with Morgan fingerprints could achieve a higher score on mean with relative lower STD, selected as the best model (Table S5, Supporting Information). In order to further improve the score, grid search method was applied to obtain optimal hyper parameters. The hyper parameters were as follows:

bootstrap = True, class_weight = None, criterion = “entropy”,
max_depth = None, max_features = “auto”, max_leaf_nodes = None,
min_impurity_decrease = None, min_impurity_split = 1e‐07,
min_samples_leaf = 1, min_samples_split = 2,
min_weight_fraction_leaf = 0.0, n_estimators = 200, n_jobs = 1,
oob_score = False, random_state = 100, verbose = 0, warm_start = False
The RF model with these hyper parameters was used for prediction.

##### Dataset for Prediction

Two miRNA–mRNA pairs were used in the study, miR‐214‐ATF4 and miR‐214‐TRAF3. For each miRNA–mRNA pair, they were first joined and treated as a single RNA sequence, and then paired with each of the small molecules in database; features were generated based on the above methods. Two sets of miRNA–mRNA–small molecule data were generated. The number of records in each set of data is same with number of small molecules. These two data sets were screened against the prediction model separately, to find small molecules that can either bind with miR‐214‐ATF4 or with miR‐214‐TRAF3, respectively.

##### Choice of the Secondary Structure Prediction Method

Three kinds of prediction tools (RNAfold, Mfold and AveRNA) were employed to compare the difference in miRNA–mRNA secondary structure prediction in dot‐bracket format. Five kinds of miRNA–mRNA sequences were randomly selected.

##### Verification for miRNA–mRNA Interaction as a Single RNA Sequence

Using the RNAfold as the secondary structure prediction method, five random kinds of miRNA–mRNA sequences were selected. Different placeholders were set in the binding site of the mRNA–miRNA sequences; 0 placeholder type indicated the input was the original mRNA–miRNA sequence. The other two random placeholders indicated mRNA and miRNA were divided separately.

##### Supplement of the Source Code and Data Set

The source code and training set including the feature values of the predictive model were supplemented: https://github.com/wanyy063700/SMTRS.

##### Wild Type and Mutant RNA Loop Synthesis

RNA and its mutants were synthesized on a 1 µm scale on a K&A H8 standard DNA/RNA synthesizer using commercially available 5′‐*O*‐DMT‐2′‐*O*‐TBDMS nucleoside (ABz, CAc, GAc, and U) phosphoramidite monomers.^[^ ^33^ ^]^ All oligonucleotides were synthesized in DMT‐off mode. For binding assays leading to affinity ranking, the RNAs were biotinylated at their 5′‐end using biotin‐TEG (Glen Research), allowing immobilization onto SA‐coated beads. After completion of the coupling reactions, the solid support was suspended in ammonium hydroxide/methylamine solution (prepared by mixing one volume of ammonium hydroxide [28%] with one volume of 40% aqueous methylamine) and heated at 65 °C for 15 min to release the product from the support and to complete the removal of all protecting groups except the TBDMS group at the 2′‐position. The solid support was filtered, and the filtrate was concentrated to dryness. For 2′‐*O*‐TBDMS RNA, the obtained residue was re‐suspended in 115 µL of anhydrous dimethyl formamide and then heated for 5 min at 65 °C to dissolve the crude product. Triethylamine (TEA, 60 µL) was added to each solution, and the solutions were mixed gently. TEA·3HF (75 µL) was added to each solution, and the tubes were then sealed tightly and incubated at 65 °C for 2.5 h. The reaction was quenched with 1.75 mL of DEPC‐treated water. Following deprotection, the oligonucleotides were desalted/ buffer exchanged into ddH_2_O and lyophilized to dryness. Purification was performed on an Agilent 1200 reverse‐phase high‐performance liquid chromatography (HPLC) to yield the final products. Mass of the RNAs was confirmed by ESI‐MS. Folding of RNAs was achieved in ammonium acetate buffer (pH 7.4) or in binding buffer by heating the RNA oligonucleotides at 90 °C for 2 min, followed by slow cooling to room temperature over 2 h.^[^ ^34^ ^]^

##### Cell Culture

Osteoblast‐like cells MC3T3‐E1 were obtained from ATCC, Manassas, VA, USA. The cells were grown in *α*­MEM medium supplemented with 10% fetal bovine serum (FBS) and 1% penicillin and streptomycin (Invitrogen) in a chamber containing 5% CO_2_ and 95% humidity at 37 °C. For the experiments, confluent cells were removed using 0.25% trypsin containing 0.03% EDTA, resuspended, and plated onto six‐well plates at a density of 2 00 000 cells per well. The RAW 264.7 cell line was used for osteoclast differentiation. The cells were cultured in DMEM (Biochrom, Berlin, Germany) containing 10% heat‐inactivated FBS and 20 U penicillin/20 µg mL^−1^ streptomycin (Biochrom). For differentiation, 30000 RAW264.7 cells per well were plated in 24‐well plates containing 40 ng mL^−1^ of mouse RANKL. The medium was changed every 2–3 days.

##### Real‐Time PCR

Total RNA from cells was extracted with TRIzol Reagent (Invitrogen) and treated with DNase I (Ambion) at 37 °C for 30 min. 1 µg of RNA was reverse transcribed using a High Capacity cDNA Reverse Transcription Kit (Applied Biosystems) and used in real time‐PCR.^[^ ^35^ ^]^

##### Western Blot

After treatment with a series concentrations of the small molecules, cells were collected and lysed in lysis buffer (50 mm Tris, pH 7.5, 250 mm NaCl, 0.1% SDS, 2 mm dithiothreitol [DTT], 0.5% NP‐40, 1 mm PMSF, and protease inhibitor cocktail) on ice for 30 min. Protein fractions were collected by centrifugation at 15000 *g* at 4 °C for 10 min and then subjected to SDS‐PAGE and transferred to polyvinylidene difluoride membranes. The membranes were blocked with 5% BSA and incubated with specific antibodies overnight. A horseradish peroxidase‐labeled secondary antibody was added and visualized using an enhanced chemiluminescence kit (Pierce).^[^ ^9^ ^]^

##### LC‐MS

LC analyses were performed using an Agilent 1100 system equipped with a quaternary pump and a refrigerated plate autosampler (Agilent Technologies). An Applied Biosystems API 3000 triple quadrupole mass spectrometer, equipped with a turbo ion spray source ionizing in the negative mode, was used to obtain the mass spectrometry data. A Phenomenex Luna C18 column (50 mm × 2.0 mm i.d., 5 µm) maintained at 40 °C was used for chromatographic separation.^[^ ^36^ ^]^

##### ITC

Isothermal calorimetric measurements were performed at 25 °C on a MicroCal iTC200 instrument (GE Health Care/Microcal, Northampton, MA, USA). miR‐214‐ATF4 loop and miR‐214‐TRAF3 loop, respectively, were injected into a 200 µL calorimetric cell and titrated against 0.5 mmol L^−1^ of small molecule OB‐4 and OC‐3, respectively, in a 40 µL syringe at 25 °C under constant stirring at 500 rpm. The resulting thermograms were analyzed with one set of binding site models using Microcal Origin 7.0.^[^ ^37^ ^]^

##### SPR Assay

SPR experiments were performed at 25 °C with a Biacore X‐100 apparatus (Biacore GE). The biotin‐labeled miR‐214‐ATF4 and miR‐214‐TRAF3 loop diluted with the running buffer were injected and were immobilized on a streptavidin‐derivatized gold chip (SA chip from Biacore) with a flow rate of 1 µL min^−1^ until ≈300 RU were reached. Direct binding of small molecules OB‐4 and OC‐3, respectively, was measured by injection of increasing concentrations of each small molecule OB‐4 and OC‐3, respectively, over the immobilized loop surfaces at a flow rate of 50 µL min^−1^ for a period of 60 s followed by a dissociation period of 120 s. Regeneration of the surface was made with NaCl 200 mm/NaOH 10 mm using a flow rate of 10 µL min^−1^ during 30 s.^[^ ^38^ ^]^

##### Cell Viability Assay (MTT, 3‐(4,5‐dimethyl‐2‐thiazolyl)‐2,5‐diphenyl‐2‐H‐tetrazolium bromide)

Cells were inoculated into 96‐well plates at a density of 5 × 10^3^ per well per 100 µL and cultured overnight. Small molecules with indicated concentrations were added to each well for 72 h. After incubation, 10 µL MTT solution (5 mg mL^−1^ dissolved in PBS, pH 7.4) was added to each well for an additional 4 h. After discarding the culture medium, 100 µL DMSO was added to each well for 10 min to sufficiently dissolve formazan crystals. The optical density (OD) was evaluated using a DTX 880 Multimode Detector (Beckman, USA), with a detection wavelength at 595 nm.^[^ ^39^ ^]^

##### Alkaline Phosphatase Staining

Alkaline phosphatase staining was monitored using a fast violet B salt kit (Sigma Aldrich). Briefly, one fast violet B salt capsule was dissolved in 48 mL distilled water and 2 mL naphthol AS‐MX phosphate alkaline solution. Cells were fixed by immersion in a citrate‐buffered acetone solution (two parts citrate and three parts acetone) for 30 s and rinsed in deionized water for 45 s. The samples were then incubated with alkaline phosphatase stain for 30 min. The whole procedure was protected from light. After rinsing in deionized water for 2 min, samples were counterstained with Mayer's hematoxylin solution for 10 min. The staining was evaluated microscopically.^[^ ^40^ ^]^

##### TRAP Staining

The differentiated RAW 264.7 cells were washed with PBS and treated with 4% paraformaldehyde solution for 10 min at room temperature. After washing with PBS, the cells were stained with TRAP‐staining solution (50 mm sodium tartrate, 45 mm sodium acetate, 0.1 mg mL^−1^ naphthol AS‐MX phosphate (Sigma‐Aldrich), and 0.6 mg mL^−1^ fast red violet LB salt [Sigma‐Aldrich], pH 5.2) for 1 h at room temperature. TRAP‐positive cells that contained three or more nuclei were determined to be multinuclear osteoclasts.^[^ ^41^ ^]^

##### Lentivirus Production and Infection

The pre‐miR‐214 sequence was amplified and cloned into the pCDH‐CMV‐MCS‐EF1‐coGFP lentiviral vector (System Biosciences, California, USA). Virus packaging and infection were performed according to standard protocols as recommended by the manufacturer. The packaged lentiviruses were named Lv‐miR214. The empty lentiviral vector was used as a control. The primers are the following: pre‐miR‐214, 5′‐ATAGAATTCTTTCTCCCTTTCCCCTTACTCTCC‐3′ (forward) and 5′‐CCAGGATCCTTTCATAGGCACCACTCACTTTAC‐3′ (reverse).^[^ ^42^ ^]^

##### AGO2–Small Molecule Interaction Assay

Briefly, cells (1.5  ×  10^7^) were suspended in 0.3 mL of miLysis buffer, supplemented with protease and RNase inhibitors, after incubation on ice for 10 min and one freeze‐thaw cycle; the lysate was diluted five times with lysis buffer, and the cytoplasmic fraction was isolated by centrifugation at 12000 × *g* at 4 °C for 5 min. To eliminate nonspecific binding, the lysate was incubated with protein A/G‐agarose beads (SantaCruz) at 4 °C for 1 h. The precleared lysates were then mixed with mouse anti‐AGO2 (15 µg of Ab/mg of lysate) armed beads. After incubation overnight at 4 °C on a rocking platform, AGO2‐IP beads were washed three times with ice cold wash buffer.^[^ ^43^ ^]^ To determine the level of small molecules, AGO2‐IP beads were heated at 95 °C for 10 min and then LC‐MS performed. To determine the level of miR‐214, AGO2‐IP beads were heated at 95 °C for 10 min and then RIP‐Assay Kit for microRNA (MBL International Corporation) performed.

##### Generation of Transgenic Mice with Osteoblast‐Specific Expression of miR‐214

The generation of transgenic osteoblast‐specific miR‐214 gene expression founder mice was established in the previous work.^[^ ^9^ ^]^ In brief, pre‐miR‐214 cDNA driven by BGLAP promoter was constructed. The fragments of the BGLAP‐pre‐miR‐214 were purified and then introduced into oocytes of C57BL/6J F2 mouse by microinjection. The oocytes were then surgically implanted into pseudopregnant C57BL/6J dams. The transgenic mice were subsequently maintained at Hong Kong Baptist University.

##### Preparation of the (AspSerSer)_6_‐Liposome Encapsulating the Small Molecule OB‐4

The lyophilization/rehydration method was employed to entrap the small molecule OB‐4 with liposomes. First, the liposomes were prepared by lipid film method described as previous study.^[^ ^44^ ^]^ Briefly, the lipids of 1,2‐dioleoyl‐3‐trimethylammonium‐propane (DOTAP), dioleoylphosphatidylethanolamine (DOPE), cholesterol (Chol), DSPE‐mPEG2000, and DSPE‐PEG2000‐MAL at a molar ratio of 42:15:38:3:2 dissolved in chloroform were dried into a thin film and hydrated with 10 mm phosphate buffer saline (PBS, pH 7.4) pre‐incubated in water bath at 50 °C to form multilamellar vesicles (MLVs). The resulting MLVs were then extruded in a LipoFast mini extruder (Lipofast, Avestin, Toronto, Canada) through two stacked polycarbonate membranes of 0.2 and 0.1 µm in a stepwise manner with five cycles, respectively, to form large unilamellar vesicles (LUVs). Then, (aspartate‐serine‐serine)_6_ with an N‐terminal acetylcysteine residue (ChinaPeptides CO., Ltd, China) was incubated with preformed liposome for 2 h at ambient temperature. The molar ratio of (AspSerSer)_6_ to DSPE‐PEG2000‐MAL was 3:1. Subsequently, the liposome suspension was purified by size exclusion chromatography with Sepharose CL‐4B column to remove the un‐conjugated (AspSerSer)_6_. The quantification of cholesterol was conducted with Infinity Cholesterol Liquid Stable Reagent (Thermo Electron; Melbourne, Australia) to assess the lipid concentration. The liposome without (AspSerSer)_6_ as control was prepared using DSPE‐mPEG2000 instead of DSPE‐PEG2000‐MAL. The liposome suspensions in 0.5 mL aliquots were mixed with 0.5 mL distilled water containing mannitol (molar ratio of mannitol‐to‐lipid = 5) and lyophilized for 48 h using freeze‐dryer (Labconco, Freezezone 6, USA). Finally, the above lyophilized liposomes with 15 mmol lipids were rehydrated by adding 0.5 mL DEPC‐treated water containing the small molecule OB‐4 and were incubated for 20 min at room temperature. The entrapment procedure was performed immediately before use and then sterilized by passing through a 0.22 mm sterile filter.

##### Treatment Protocols of Small Molecule OB‐4 in Mice

Six TG‐214 mice and wild type (WT) mice were euthanized before the treatment as the baseline (TG‐214 BS and WT BS). Four‐week‐old mice were given (AspSerSer)_6_‐liposome plus OB‐4 (10 mg kg^−1^) (dissolved in saline for application), (AspSerSer)_6_‐liposome plus de‐hydroxylated OB‐4 (10 mg kg^−1^), the negative control group was treated with an equal volume of (AspSerSer)_6_‐liposome by tail vein injection, at a frequency of two injections a week (*n* = 6 for each group). At 4 weeks after the injection, all mice were euthanized. Before euthanasia, all the mice were injected intraperitoneally with calcein green (10 mg kg^−1^ body weight) in a time sequence of 10 and 2 days, respectively. After euthanasia, the femurs were collected for micro‐CT and histomorphometric analysis. All experiments were performed in accordance with relevant guidelines and regulations, and all experimental procedures were approved by the Committees of Animal Ethics and Experimental Safety of Hong Kong Baptist University.

##### Generation of Knock‐In Mice with Osteoclast‐Specific Expression of miR‐214

The procedure of OC‐miR‐214 mice generation was thoroughly described in the previous protocols.^[^ ^10^ ^]^ First, the Rosa26‐PCAG‐STOPfl‐mmu‐miR‐214‐3p‐knock‐in mice were generated. In brief, a cassette containing the following components was constructed to target the Rosa26 locus: FRT‐LoxP‐stop codons‐three SV40 poly(A) sequences‐LoxP‐mmu‐miR‐214‐3p‐WPRE‐bGH poly(A)‐AttB‐PGK promoter‐FRT‐Neo‐PGK poly(A)‐AttP. The targeting vector was constructed, fully sequenced, and electroporated into C57BL/6 embryonic stem cells. PCR and Southern blotting were then performed to identify the positive targeting clones. Following standard procedures, we microinjected the targeted embryonic stem clones into BALB/c blastocysts in order to obtain chimeric mice. Next, the chimeric mice were intercrossed with C57BL/6 mice to obtain F1 heterozygote mice and then backcrossed with C57BL/6 mice to increase the number of heterozygote Rosa26‐PCAG‐STOPfl‐mmu‐miR‐214‐knock‐in mice. Second, we crossed the Rosa26‐PCAG‐STOPfl‐mmu‐miR‐214‐knock‐in mice with Ctsk‐Cre mice to get OC‐miR‐214 mice. The littermates were used as WT control.

##### Preparation of the D‐Asp_8_ Moiety Modified Liposome Encapsulating Small Molecule OC‐3

The lyophilization/rehydration method was employed to encapsulate small molecule OC‐3 in liposomes. First, the lipids of 1,2‐dioleoyl‐3‐trimethylammonium‐propane (DOTAP), dioleoylphosphatidylethanolamine (DOPE), cholesterol (Chol), DSPE‐mPEG2000, and DSPE‐PEG2000‐MAL at a molar ratio of 42:15:38:3:2 dissolved in chloroform were dried into a thin film and hydrated with 10 mm phosphate buffer saline (PBS, pH 7.4) pre‐incubated in water bath at 50 °C to form MLVs. The resulting MLVs were then extruded in a LipoFast mini extruder (LipoFast, Avestin, Toronto, Canada) through two stacked polycarbonate membranes of 0.2 and 0.1 mm in stepwise manner with five cycles, respectively, to form LUVs. Then, the D‐Asp_8_ peptide with a C‐terminal sulfhydryl residue (ChinaPeptides CO., Ltd, China) was incubated with preformed liposome for 2 h at ambient temperature. The molar ratio of D‐Asp_8_ moiety to DSPE‐PEG2000‐MAL was 2:1. Subsequently, the liposome suspension was purified by size exclusion chromatography with Sepharose CL‐4B column to remove the un‐conjugated D‐Asp_8_ moiety. The quantification of cholesterol was conducted with Infinity Cholesterol Liquid Stable Reagent (Thermo Electron, Melbourne, Australia) to assess the lipids concentration. The liposome conjugated without D‐Asp_8_ moiety as control was prepared using DSPE‐mPEG2000 instead of DSPE‐PEG2000‐MAL. The liposome suspension in 0.5 mL aliquots were mixed with 0.5 mL distilled water containing mannitol (molar ratio of mannitol‐to‐lipid = 5:1) and lyophilized for 48 h using freeze‐dryer (Labconco, Freezezone 6, USA). Finally, the above lyophilized liposomes with 15 mmol lipids were rehydrated by adding 0.5 mL DEPC‐treated water containing small molecule OC‐3 and were incubated for 20 min at room temperature. The encapsulation procedure was performed immediately before use and then sterilized by passing through a 0.22 mm sterile filter.

##### Treatment Protocols of Small Molecule OC‐3 in Mice

Six OC‐214 mice and wild type mice were euthanized before the treatment as baseline (OC‐214‐BS and WT‐BS). Four‐week‐old mice were given (d‐Asp_8_)‐liposome plus OC‐3 (20 mg kg^−1^) (dissolved in saline for application), (d‐Asp_8_)‐liposome plus methylated OC‐3 (20 mg kg^−1^), the negative control group was treated with an equal volume of (d‐Asp_8_)‐liposome by tail vein injection, at a frequency of two injections a week (*n* = 6 for each group). At 4 weeks of injection, all mice were euthanized. Before euthanasia, all the mice were injected intraperitoneally with calcein green (10 mg kg^−1^ body weight) in a time sequence of 10 and 2 d, respectively. After euthanasia, the right femurs were collected for micro‐CT. The left femora were collected for histomorphometric analysis. All experiments were performed in accordance with relevant guidelines and regulations and all experimental procedures were approved by the Committees of Animal Ethics and Experimental Safety of Hong Kong Baptist University.

##### ELISA

The bone resorption marker CTX‐1 level in the serum was quantified in mice using commercially available ELISA kits (Uscn Life Science Inc., Wuhan, China) according to the manufacturer's instructions and the previously published protocol.^[^ ^9^ ^]^ The determination was performed in duplicate, and the plates were analyzed using an automatic microplate reader (Thermo Scientific, USA).

##### MicroCT Analysis

MicroCT system (vivaCT40, SCANCO Medical, Switzerland) was adopted to scan the distal femur and proximal tibia ex vivo, with details described in the established protocols.^[^ ^9^ ^]^ In brief, 423 slices with a voxel size of 10 µm were scanned at the region of the distal femur and proximal tibia from the growth plate. Eighty continuous slices beginning at 0.1 mm from the most proximal aspect of the growth plate in which both condyles were no longer visible were adopted for analysis. The whole trabecular bone was segmented for 3D reconstruction (sigma = 1.2, supports = 2, and threshold = 180) to generate the parameters BMD and bone volume/total volume (BV/TV).

##### Bone Histomorphometric Analysis

Following our previous protocols,^[^ ^9^ ^]^ the distal femur and proximal tibia were dehydrated in graded concentrations of ethanol and embedded without decalcification in modified methyl methacrylate. After that, frontal sections of trabecular bone were obtained from the distal femur or proximal tibia at 10 µm thickness using a EXAKT Cut/Grinding System (EXAKT Technologies, Inc. Germany). Bone dynamic histomorphometric analysis were performed to measure the trabecular bone formation rate (BFR/BS) and trabecular bone mineral apposition rate (MAR) at distal femur for evaluation of bone formation using fluorescence microscope (Leica image analysis system, Q500MC). For proximal tibia, TRAP staining of the obtained bone sections were performed followed by bone histomorphometric analyses. The percentage of trabecular bone surface covered by osteoclasts (Oc.S/BS) and osteoclast number per bone perimeter (N.Oc/b.Pm) was used for evaluation of bone resorption activity. These two parameters were obtained from microscope (Leica image analysis system, Q500MC) and then analyzed by Image J (NIH, USA) and BIOQUANT OSTEO analysis software (Nashville, TN, USA).^[^ ^10^ ^]^

##### Statistical Analysis

All values were expressed as mean ± standard deviation. Student's *t*‐test was used for comparison between two groups. One‐way ANOVA with post hoc tests was used for multiple‐group comparisons. A statistical software program (SPSS version 19.0, IBM SPSS Statistics, USA) was used and *p* < 0.05 was considered to be statistically significant.

## Conflict of Interest

The authors declare no conflict of interest.

## Supporting information

Supporting Information
