# Supplementary material for: A Loop‐Based and AGO‐Incorporated Virtual Screening Model Targeting AGO‐Mediated miRNA–mRNA Interactions for Drug Discovery to Rescue Bone Phenotype in Genetically Modified Mice
Source: Adv Sci (Weinh). 2020 May 28;7(13):1903451. doi: 10.1002/advs.201903451 (PMC7341099; doi:10.1002/advs.201903451)
Supplement: Supplementary file 1 — Supporting Information [file ADVS-7-1903451-s001.pdf]

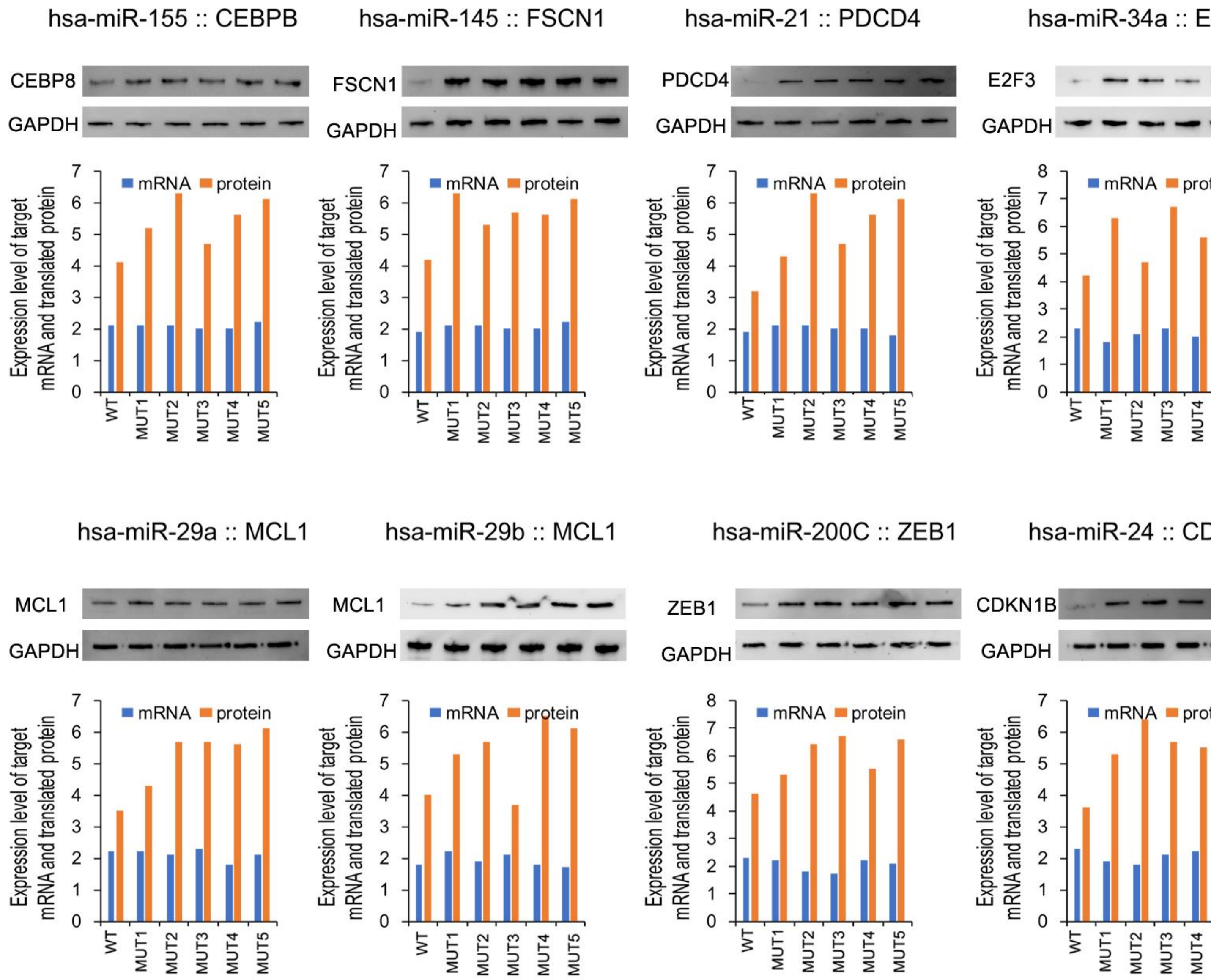

S-Figure 1. Experimental data for the functional validation of the loops in ten most widely reported miRNA-mRNA interactions. Experimental validation for the effects of the wild type and mutant loops on expression levels of target mRNAs and proteins translated by the target mRNAs, respectively.

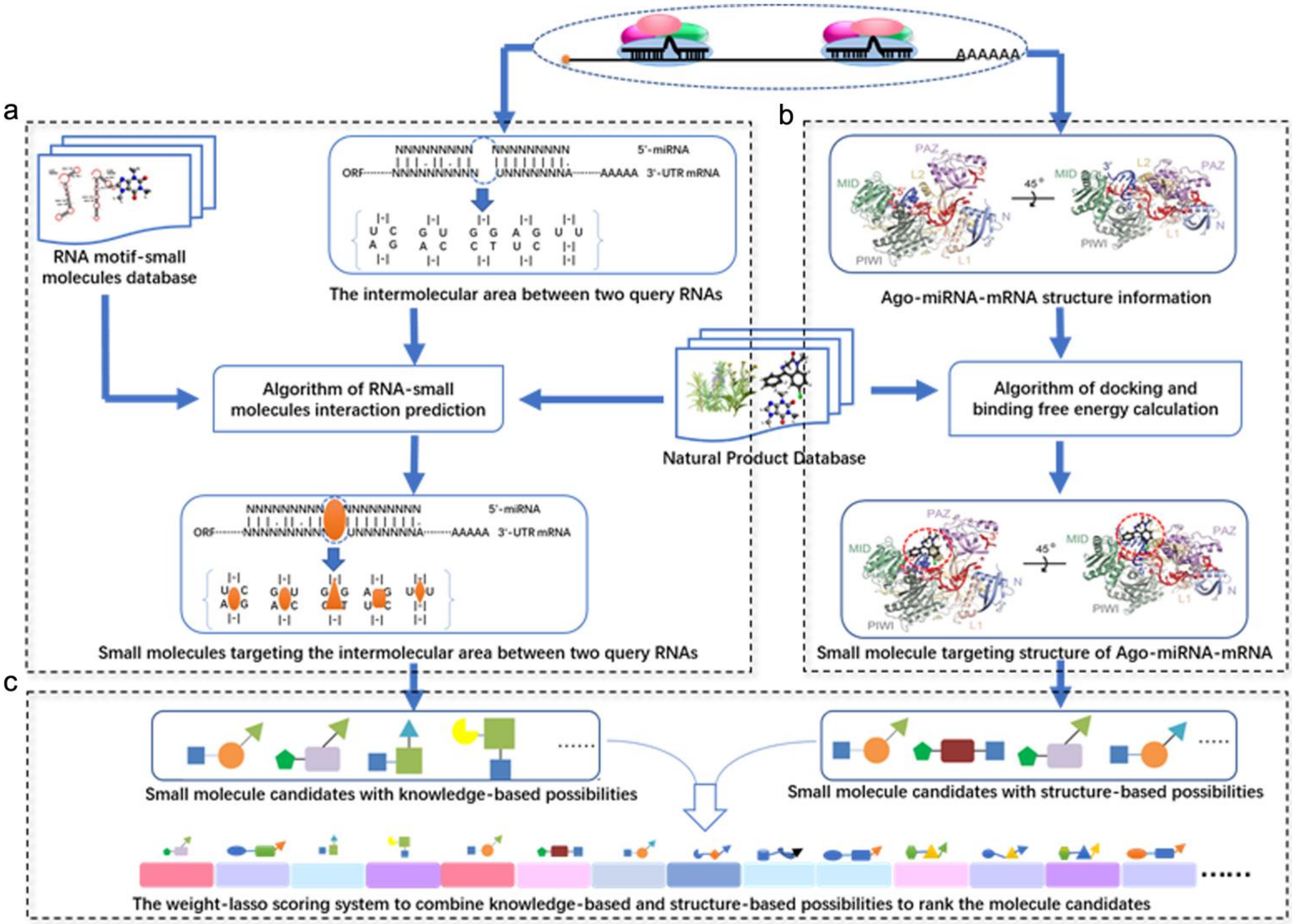

S-Figure 2. The virtual screening model by combining both knowledge-based and structure-based approaches for targeting miRNA-mRNA interaction. a) The schematic diagram of the knowledge-based virtual screening approach. b) The schematic diagram of the structure-based virtual screening approach. c) The weight-lasso scoring system to combine the knowledge-based and structure-based possibilities to rank the molecule candidates.

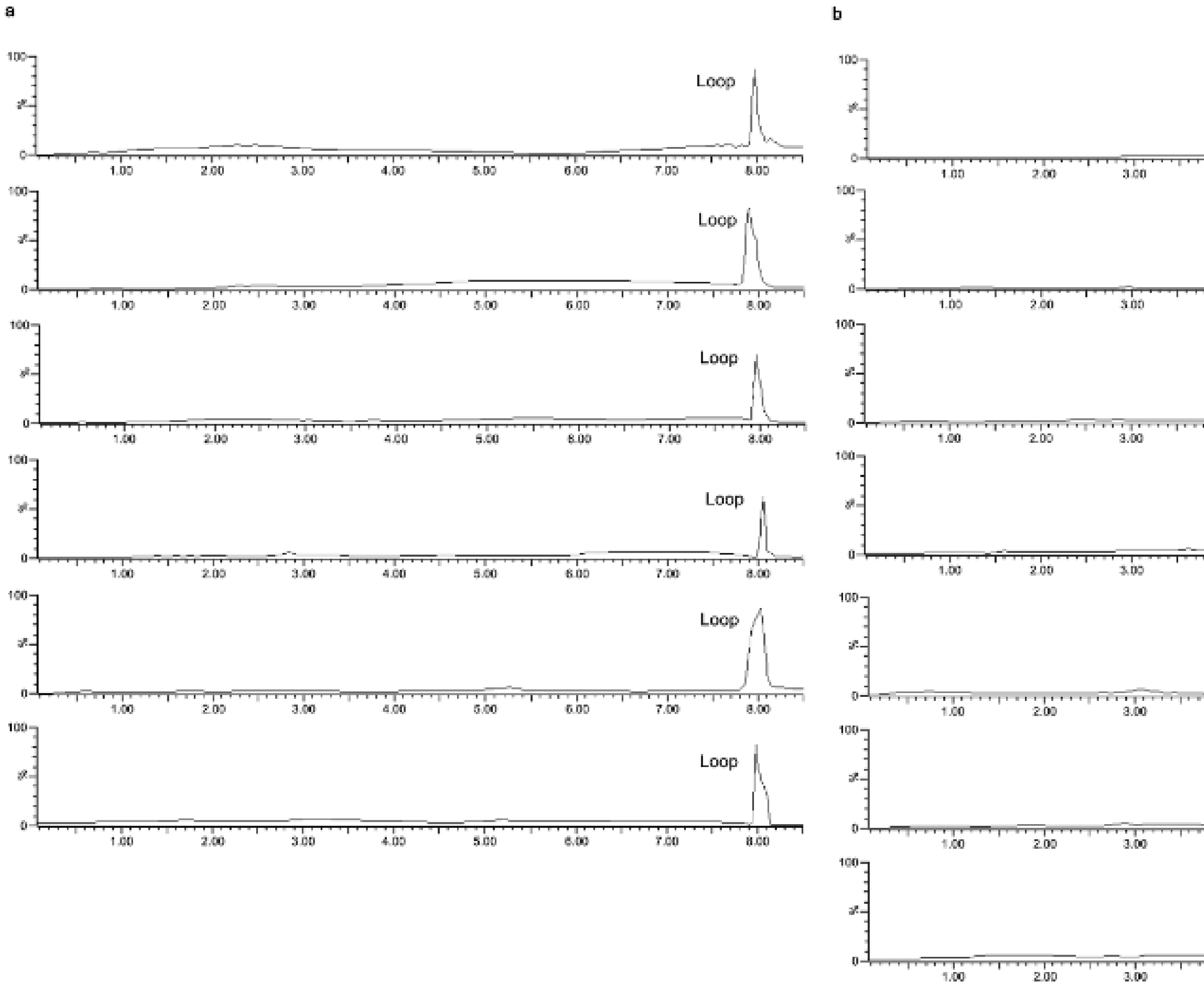

S-Figure 3. Binding assay of the small molecule candidates with the loops. a) Pull-down assay was performed using streptavidin magnetic bead to examine the interaction between biotin-miR-214-ATF4 loop and small molecule OB-5, OB-6, OB-7, OB-8, OB-9, and OB-10. Small molecule OB-5, OB-6, OB-7, OB-8, OB-9, and OB-10 captured by biotin-miR-214-ATF4 loop was examined by LC-MS, respectively (from top to bottom). b) Pull-down assay was performed using streptavidin magnetic bead to examine the interaction between biotin-miR-214-TRAF3 loop and small molecule OC-4, OC-5, OC-6, OC-7, OC-8, OC-9, and OC-10. Small molecule OC-4, OC-5, OC-6, OC-7, OC-8, OC-9, and OC-10 captured by biotin-miR-214-TRAF3 loop was examined by LC-MS, respectively (from top to bottom).

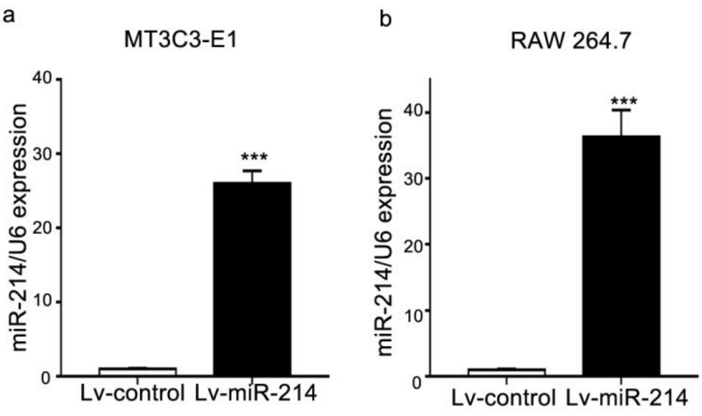

S-Figure 4. Establishment of stably expressing miR-214 MC3T3-E1 and RAW 264.7 cells. a) Expression of miR-214 in MC3T3-E1 cells infected with miR-214 lentivirus (indicated as Lv-miR-214) and control lentivirus (indicated as Lv-control) was measured by qRT-PCR. b) Expression of miR-214 in RAW 264.7 cells infected with miR-214 lentivirus (indicated as Lv-miR-214) and control lentivirus (indicated as Lv-control) was measured by qRT-PCR. ***$P < 0.001$ vs control group.

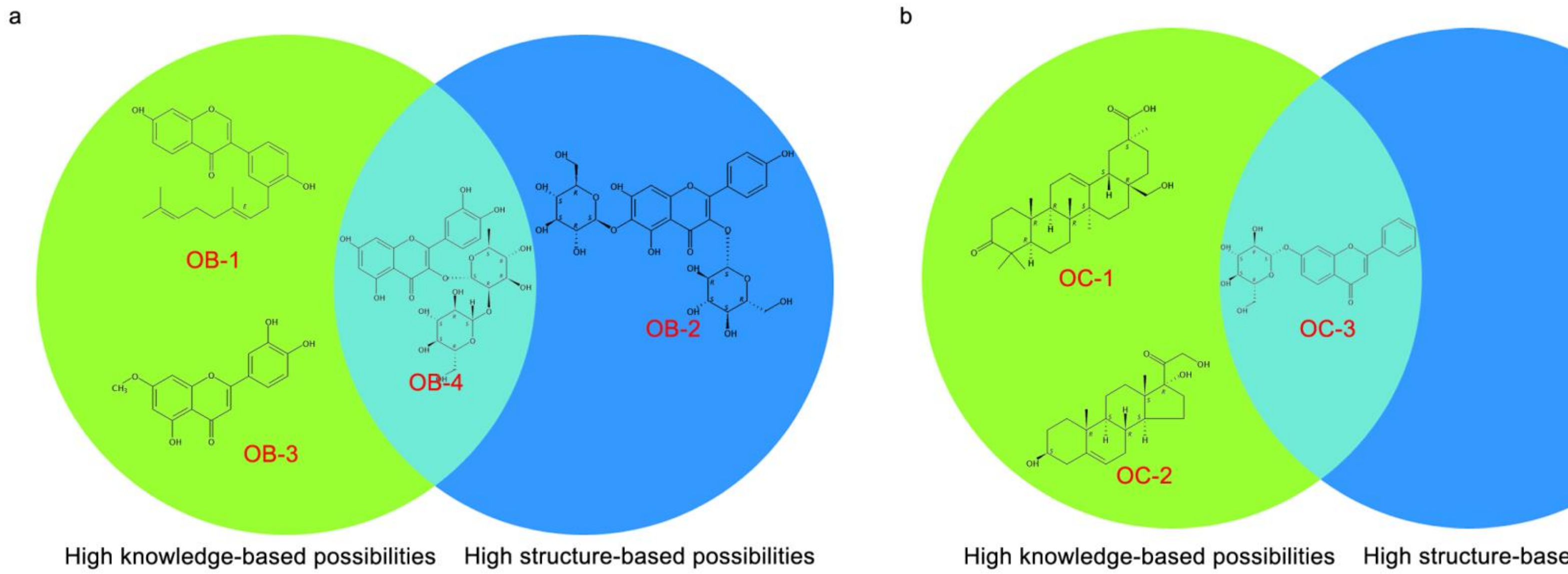

S-Figure 5. Venn diagram illustrating the overlap between the two sets of algorithms. a) Knowledge-based algorithm and the structure-based algorithm calculated scores of OB-1, OB-2, OB-3 and OB-4. b) Knowledge-based algorithm and the structure-based algorithm calculated scores of OC-1, OC-2 and OC-3.

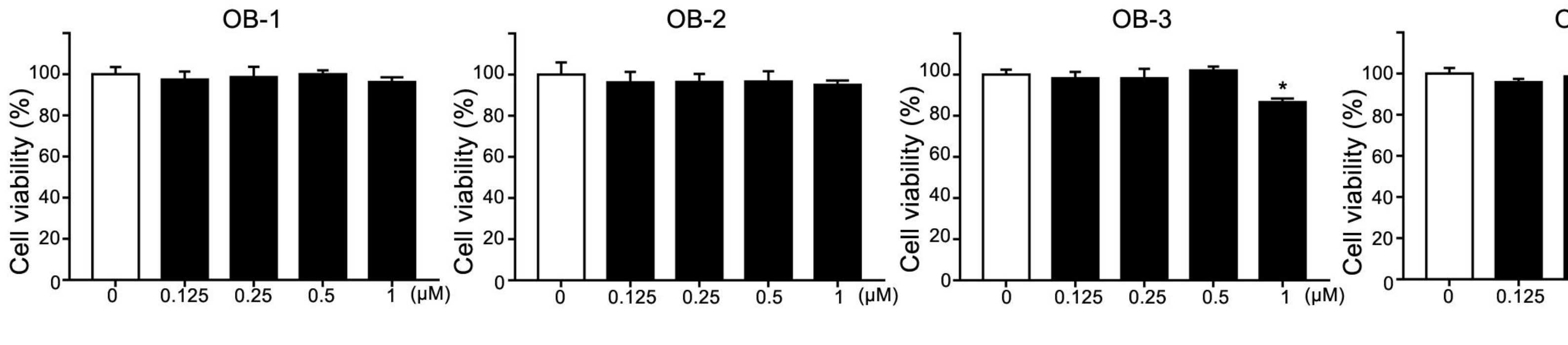

S-Figure 6. MTT assay was used to determine the non-toxic concentration of the small molecule OB-1, OB-2, OB-3, OB-4, respectively, after treatment of MC3T3-E1 cells for 72 h. *$P < 0.05$ vs control group.

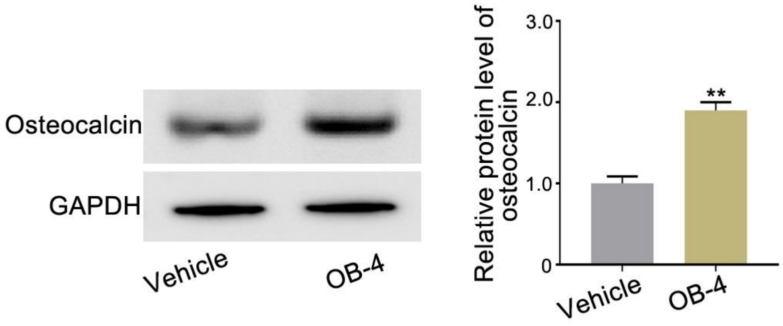

S-Figure 7. Determination of the expression of ATF4 downstream molecules after small molecule OB-4 treatment by Western blot. Representative Western blot (left) and quantification (right) of osteocalcin protein level in MC3T3-E1 cells after treatment with the small molecule OB-4 at 80 nM or vehicle for 48 h. **$P < 0.01$ vs control group.

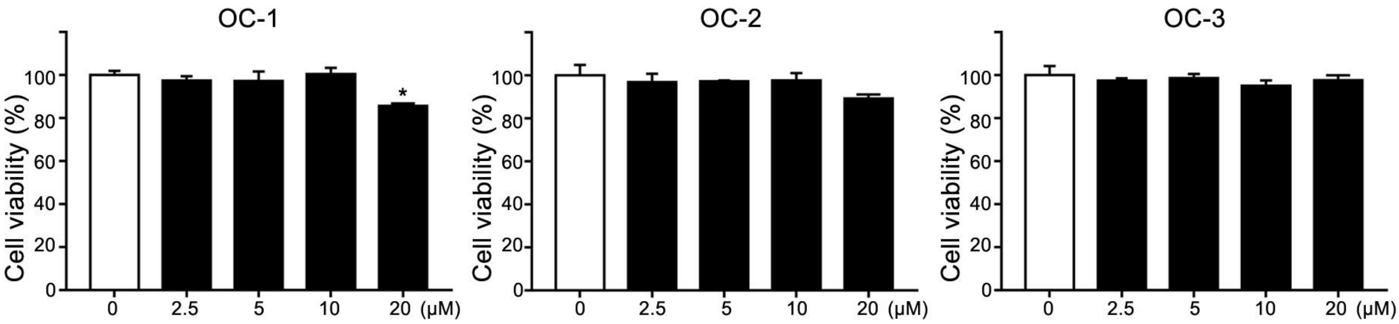

S-Figure 8. MTT assay was used to determine the non-toxic concentration of the small molecule OC-1, OC-2, or OC-3, respectively, after treatment of RAW 264.7 cells for 72 h. *$P < 0.05$ vs control group.

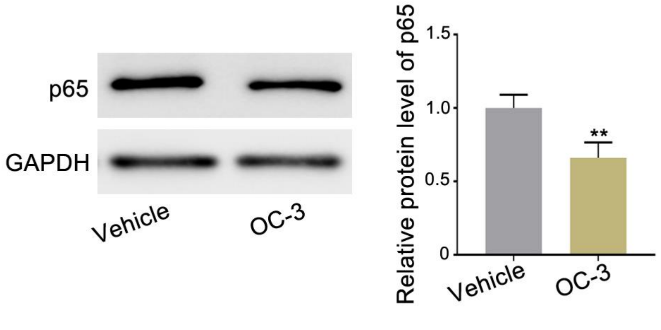

S-Figure 9. Determination of the expression of TRAF3 downstream molecules after small molecule OC-3 treatment by Western blot. Representative Western blot (left) and quantification (right) of p65 protein level in RAW 264.7 cells after treatment with the small molecule OC-3 at 6 μM or vehicle for 48 h. ***P* < 0.01 vs control group.

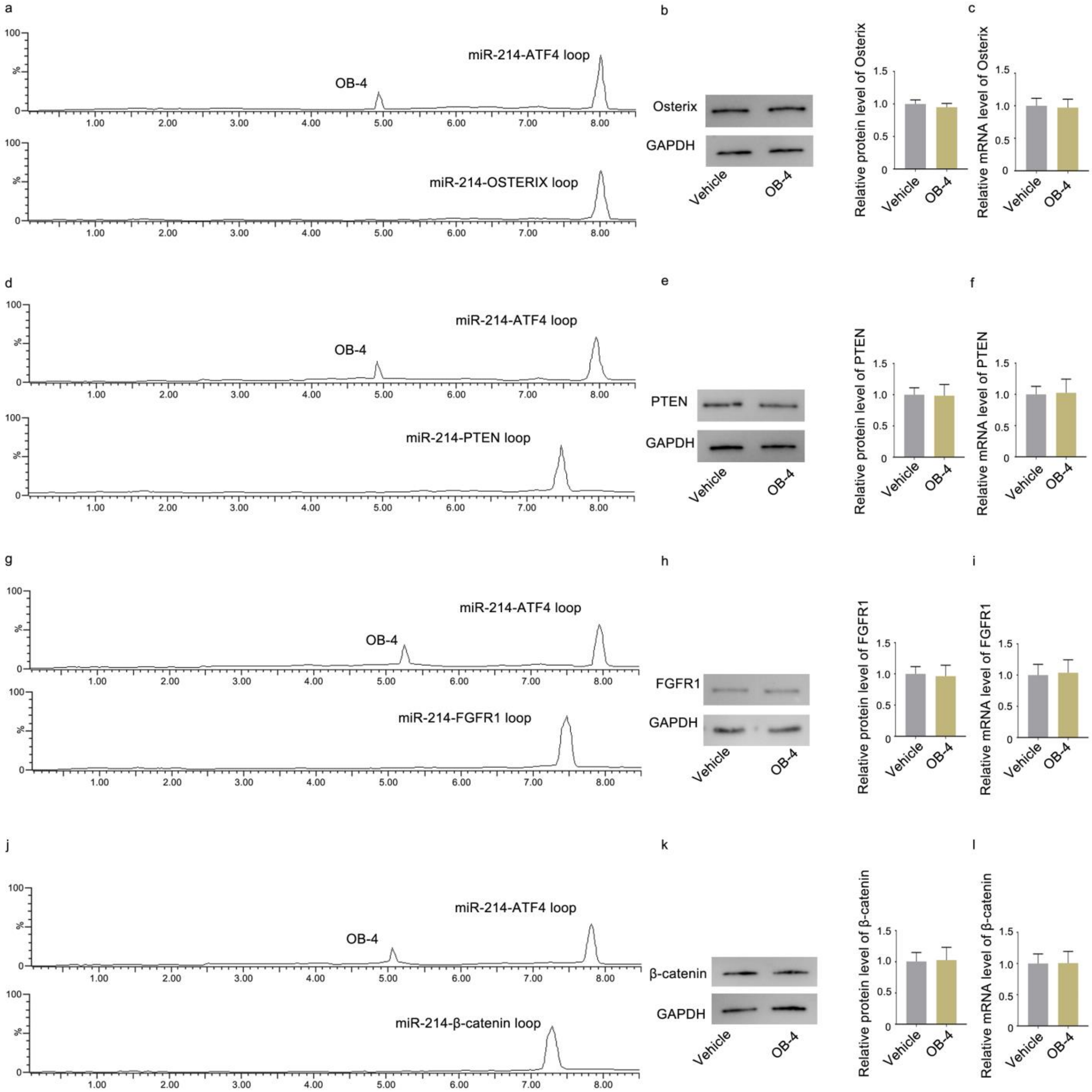

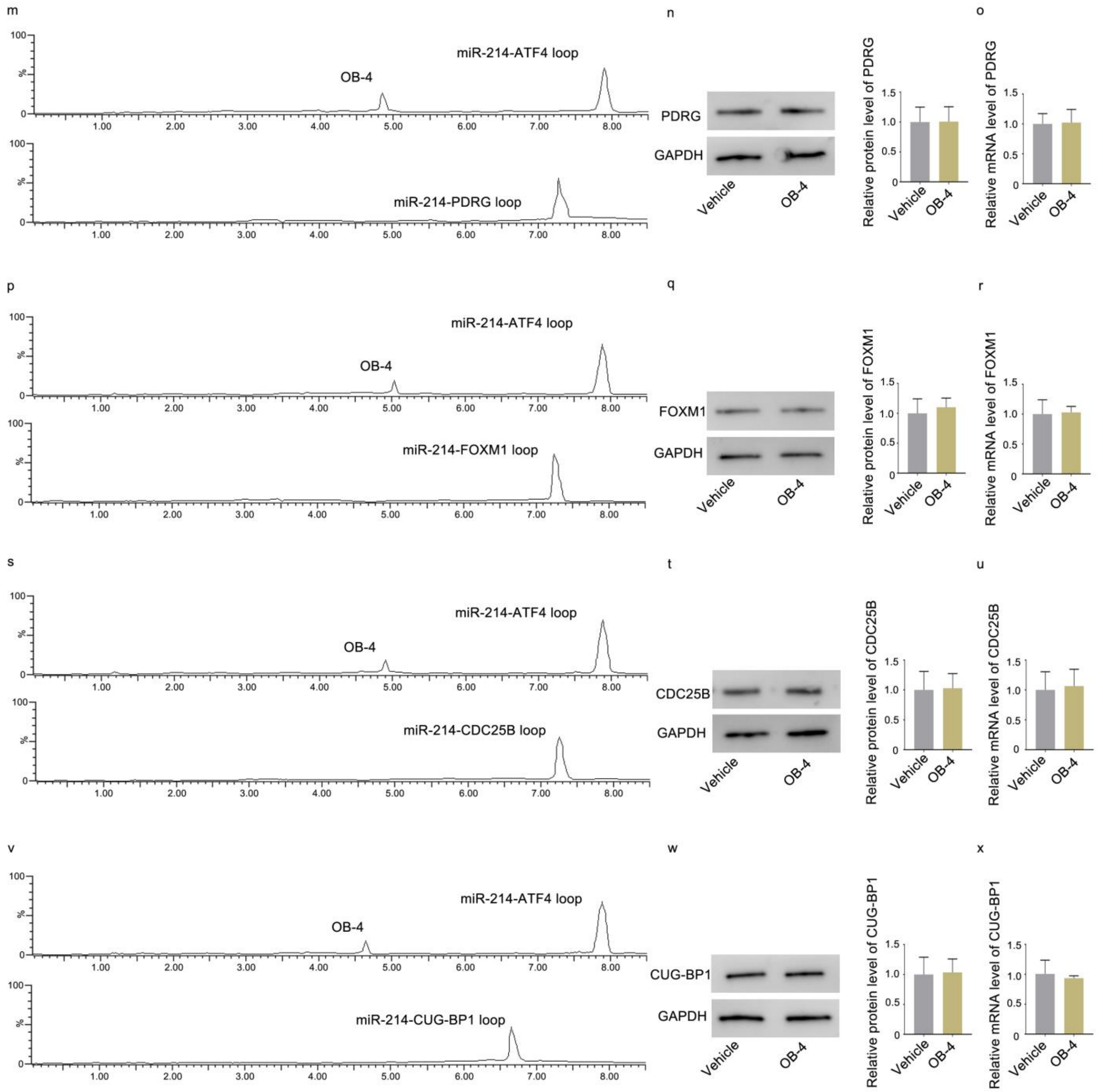

S-Figure 10. Determination of the specificity of OB-4 by LC-MS, Western blot, and PCR. a, d, g, j, m, p, s, v) Pull-down assay was performed using streptavidin magnetic bead to examine the interaction between loops formed by miR-214 and its other target mRNA, Osterix, PTEN, FGFR1, β-catenin, PDRG, FOXM1, CDC25B, CUG-BP1, and small molecule OB-4. Small molecule OB-4 captured by loops formed by miR-214 and its other target mRNA, Osterix, PTEN, FGFR1, β-catenin, PDRG, FOXM1, CDC25B, CUG-BP1, was examined by LC-MS. b, e, h, k, n, q, t, u) Representative Western blot (left) and quantification (right) of Osterix, PTEN, FGFR1, β-catenin, PDRG, FOXM1, CDC25B, CUG-BP1 protein level in MC3T3-E1 cells after treatment with the small molecule OB-4 at 80 nM or vehicle for 48 h. c, f, i, l, o, r, u, x) Real-time PCR analysis of Osterix, PTEN, FGFR1, β-catenin, PDRG, FOXM1, CDC25B, CUG-BP1 mRNA level in MC3T3-E1 cells after treatment with the small molecule OB-4 at 80 nM or vehicle for 48 h.

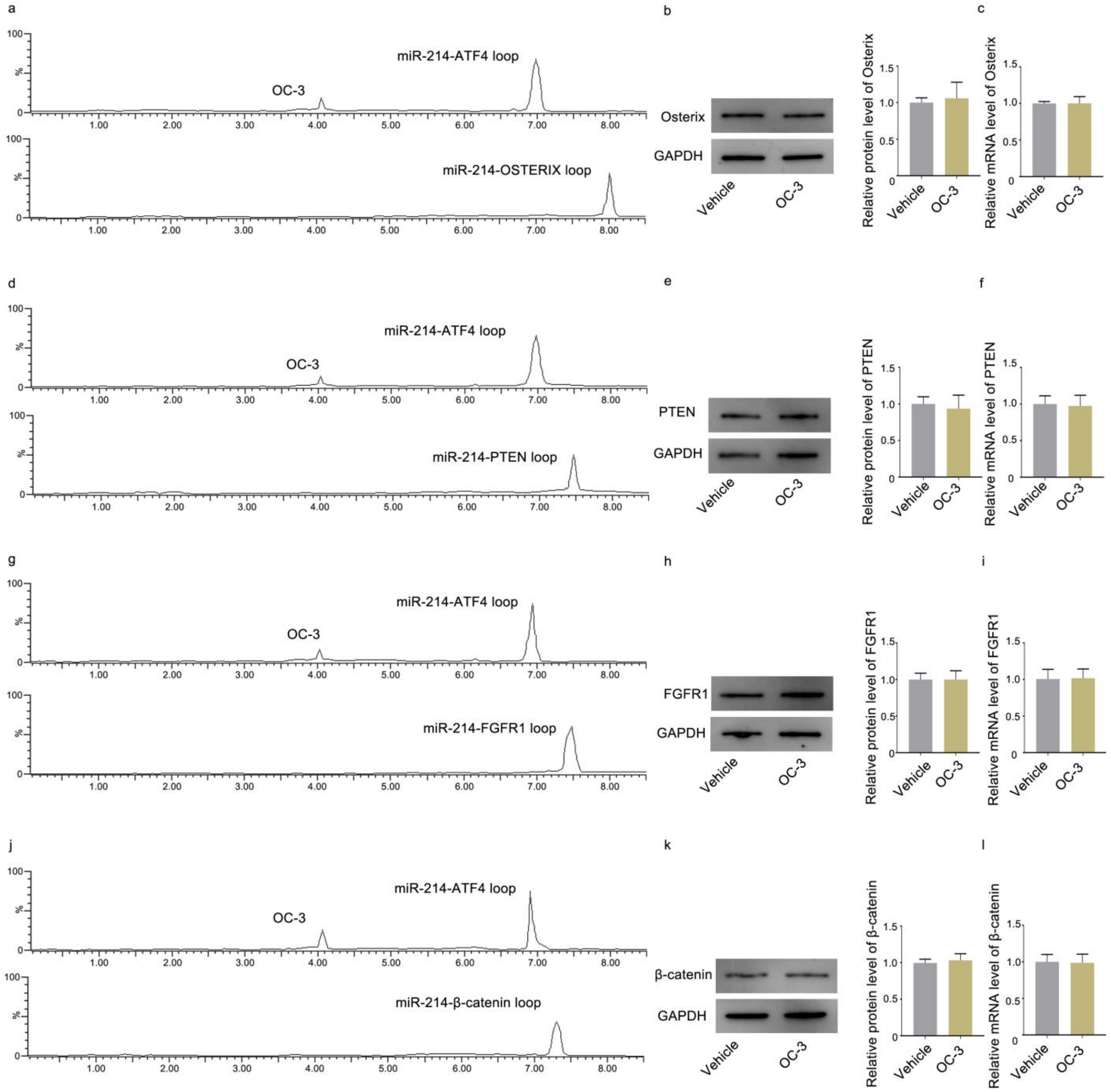
a
miR-214-ATF4 loop
OC-3
miR-214-OSTERIX loop
b
Osterix
GAPDH
Vehicle
OC-3
Relative protein level of Osterix
c
Relative mRNA level of Osterix
d
miR-214-ATF4 loop
OC-3
miR-214-PTEN loop
e
PTEN
GAPDH
Relative protein level of PTEN
f
Relative mRNA level of PTEN
g
miR-214-ATF4 loop
OC-3
miR-214-FGFR1 loop
h
FGFR1
GAPDH
Relative protein level of FGFR1
i
Relative mRNA level of FGFR1
j
miR-214-ATF4 loop
OC-3
miR-214-β-catenin loop
k
β-catenin
GAPDH
Relative protein level of β-catenin
l
Relative mRNA level of β-catenin

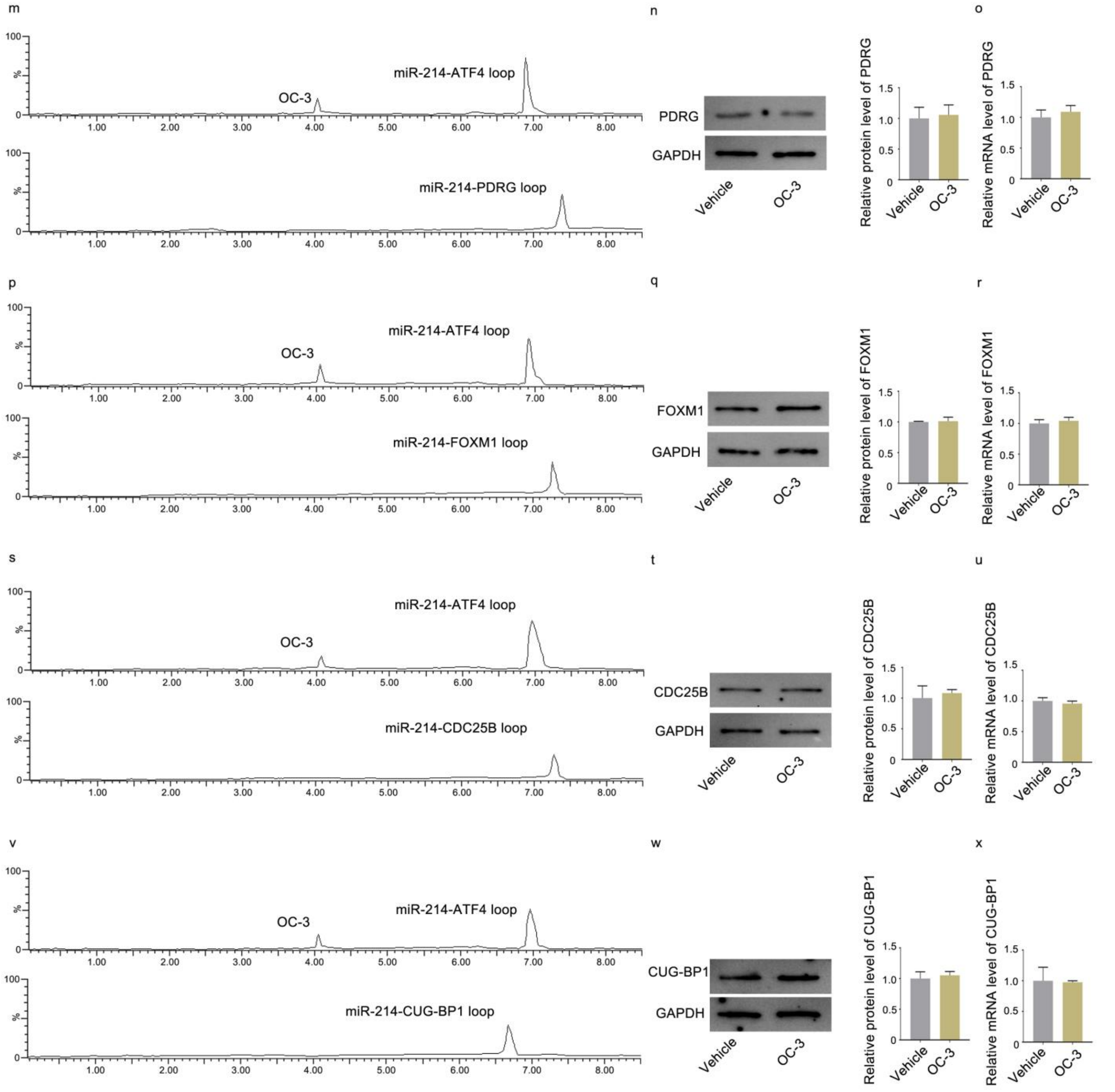

S-Figure 11. Determination of the specificity of OC-3 by LC-MS, Western blot, and PCR. a, d, g, j, m, p, s, v) Pull-down assay was performed using streptavidin magnetic bead to examine the interaction between loops formed by miR-214 and its other target mRNA, Osterix, PTEN, FGFR1, β-catenin, PDRG, FOXM1, CDC25B, CUG-BP1, and small molecule OC-3. Small molecule OC-3 captured by loops formed by miR-214 and its other target mRNA, Osterix, PTEN, FGFR1, β-catenin, PDRG, FOXM1, CDC25B, CUG-BP1, was examined by LC-MS. b, e, h, k, n, q, t, u) Representative Western blot (left) and quantification (right) of Osterix, PTEN, FGFR1, β-catenin, PDRG, FOXM1, CDC25B, CUG-BP1 protein level in RAW 264.7 cells after treatment with the small molecule OC-3 at 6 μM or vehicle for 48 h. c, f, i, l, o, r, u, x) Real-time PCR analysis of Osterix, PTEN, FGFR1, β-catenin, PDRG, FOXM1, CDC25B, CUG-BP1 mRNA level in RAW 264.7 cells after treatment with the small molecule OC-3 at 6 μM or vehicle for 48 h.

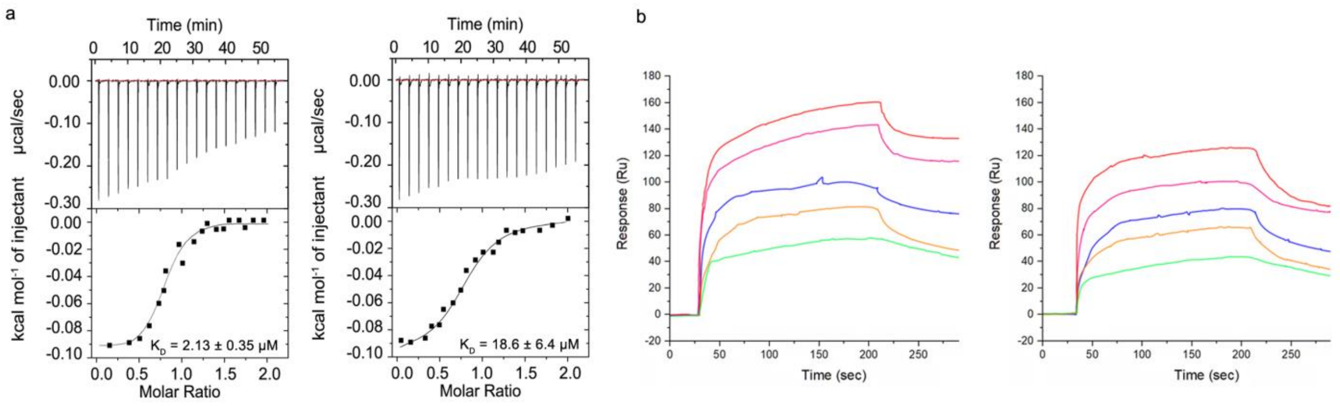

S-Figure 12. Determination of the binding of OB-4 and OC-3 to miR-214-ATF4 and miR-214-TRAF3 loop, respectively, by ITC and SPR. a) A total of 50 μM of miR-214-ATF4 loop (left) and miR-214-TRAF3 loop (right), respectively, was titrated against 0.5 mmol/L of small molecule OB-4 and OC-3, respectively. The resulting thermograms were analyzed based on the one set of binding sites model using Microcal Origin 7.0 (Microcal. Inc.). b) SPR analysis of the interaction of miR-214-ATF4 loop (left) and miR-214-TRAF3 loop (right) to small molecule OB-4 and OC-3, respectively. Small molecule OB-4 and OC-3, respectively, was added at 20 μM (red), 10 μM (pink), 5 μM (blue), 2.5 μM (orange), and 1.25 μM (green).

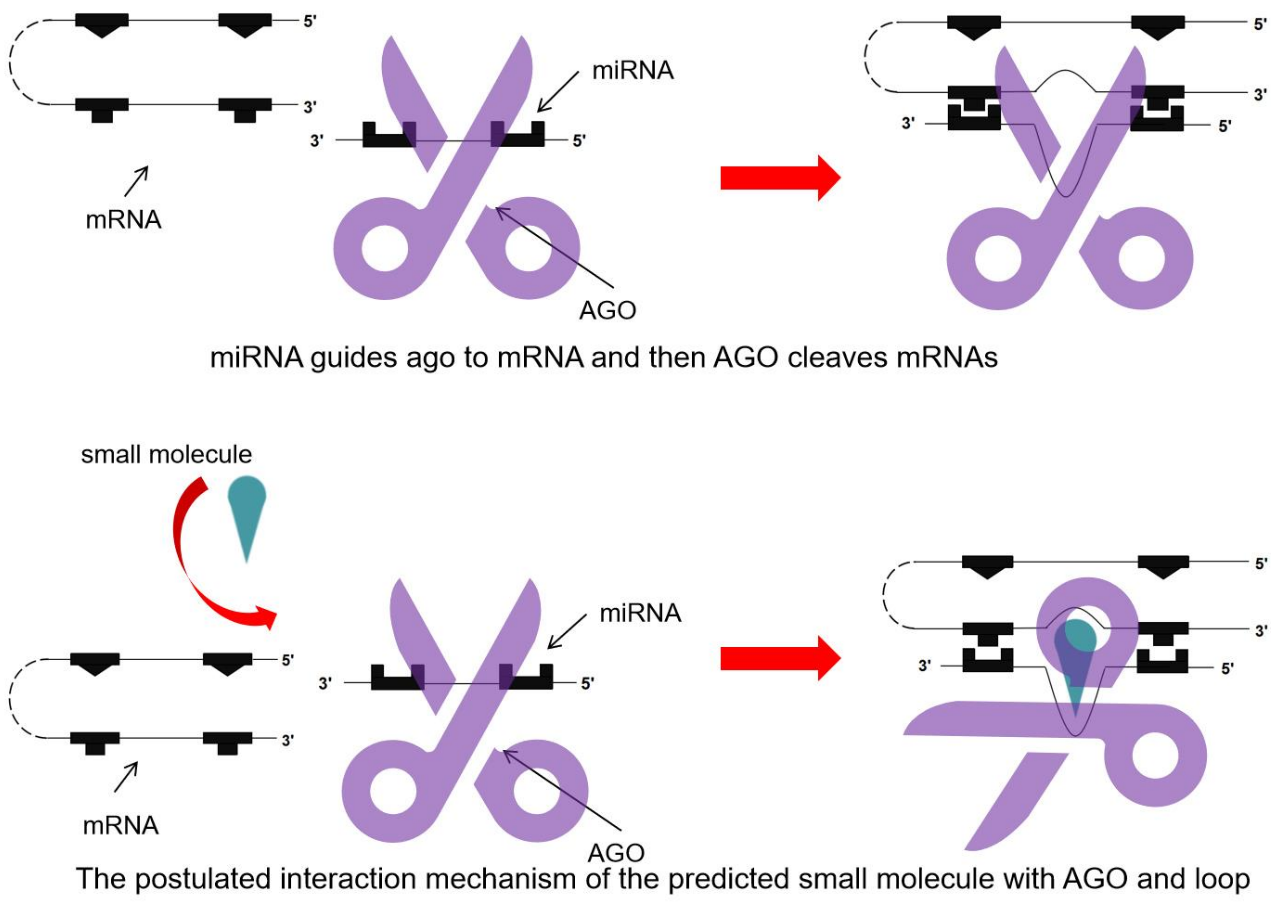

S-Figure 13. Proposed interaction mechanisms of predicted small molecule targeting the miRNA-mRNA loop. (Upper) miRNAs guide the AGO to the 3'-untranslated regions (3'-UTR) of target mRNAs and form miRNA-mRNA loops. AGO then cleaves mRNAs. (Down) The predicted small molecule could either interfere the loop formation of the miRNA with the target mRNA when

the miRNA directed the AGO to the target mRNA or directly target the AGO-mediated miRNA-mRNA loop and this targeting could sterically interfere AGO nearby to rescue the repression of mRNA translation by AGO.

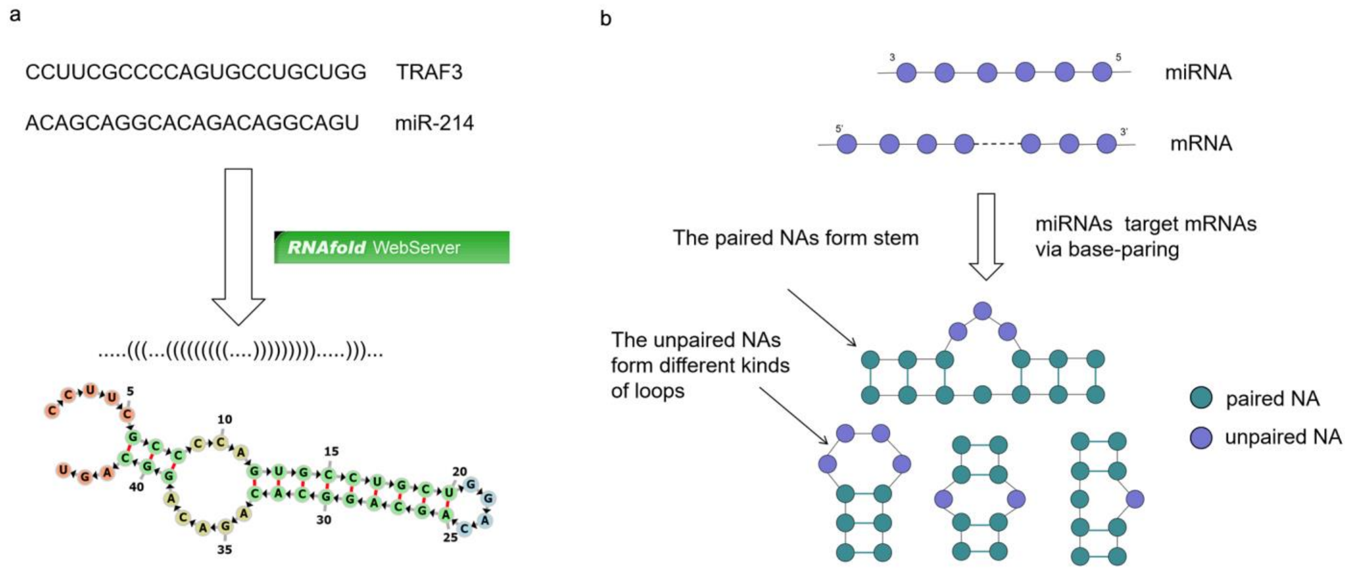

S-Figure 14. Formation of loop region. a) The miRNAs target mRNAs via base-pairing. The paired NAs form stem region, whereas the unpaired NAs form different loop regions. b) To identify the loop using our computation strategy, the sequence of the miRNA and its target mRNA was firstly input, then the unpaired bases (loops) was generated by the RNAfold program.

S-Table 1. Ten most widely reported miRNAs in meta-analysis based on PubMed library.

miRNAs	OA	WEIGHT	PVALUE	QVALUE
hsa-miR-155	CEBPB	37.8	0.000000125	0.00000075
hsa-miR-145	FSCN1	38.2	0.000000155	0.00000099
hsa-miR-21	PDCD4	38.6	0.000000185	0.00000123
hsa-miR-34a	E2F3	35.4	0.000000215	0.00000147
hsa-miR-125b	TP53INP1	32.2	0.000000245	0.00000171
hsa-miR-29a	MCL1	29.0	0.000000275	0.00000195
hsa-miR-29b	MCL1	25.8	0.000000305	0.00000219
hsa-miR-200c	ZEB1	27.5	0.000000335	0.00000243
hsa-miR-24	CDKN1B	29.2	0.000000365	0.00000267
hsa-miR-124	CDK6	30.9	0.000000395	0.00000291

S-Table 2. Ten most widely validated miRNA-mRNA interactions in meta-analysis based on PubMed library.

miRNA	Targets	RA	WB	PCR	MA	NGS	pSILAC	OT	Ref
hsa-miR-155	CEBPB	Y	Y	Y	Y	N	Y	N	27
hsa-miR-145	FSCN1	Y	Y	Y	Y	N	N	Y	27
hsa-miR-21	PDCD4	Y	Y	Y	Y	Y	N	Y	27
hsa-miR-34a	E2F3	Y	Y	Y	Y	Y	N	Y	27
hsa-miR-125b	TP53INP1	Y	Y	Y	Y	Y	N	Y	27
hsa-miR-29a	MCL1	Y	Y	Y	Y	Y	N	Y	27
hsa-miR-29b	MCL1	Y	Y	Y	Y	Y	N	Y	27
hsa-miR-200c	ZEB1	Y	Y	Y	Y	Y	N	Y	27
hsa-miR-24	CDKN1B	Y	Y	Y	Y	Y	N	Y	27
hsa-miR-124	CDK6	Y	Y	Y	Y	Y	N	Y	27

S-Table 3. Comparison of RNA secondary structure predicted by different tools in dot-bracket format.

RNA_ID	item	mRNA-miRNA sequence and predicted secondary structures
miRmR_1	mRNA-miRNA sequence	GAGACGGAGGGCCCAUGGAACUUACAGAAGAAGUUGUUGAUGAGUUCAU GGAAGAUGUCCCUAUGUCGA
	RNAfold	..((((.((((.(((((.(((((((((.........))).))))))))))).......)))).))))..
	Mfold	..((((.((((((((((.(((((((((.........))).))))))))))).....).)))).))))..
	AveRNA	..((((.((((.(((((.(((((((((.........))).))))))))))).......)))).))))..
miRmR_28	mRNA-miRNA sequence	GGGCCCGCCGCGCCAUGCCUGUGGCCGGCCC
	RNAfold	(((((.((((((.......)))))).)))))
	Mfold	(((((.((((((.......)))))).)))))
	AveRNA	(((((.((((((.......)))))).)))))
miRmR_42	mRNA-miRNA sequence	CCGCUGCUGCUGCUGCUGCUGCUGCUGCUGCUGCGG
	RNAfold	((((.((.((.((.((....)).)).)).)).))))
	Mfold	((((.((.((.((.((....)).)).)).)).))))
	AveRNA	((((.((.((.((.((....)).)).)).)).))))
miRmR_50	mRNA-miRNA sequence	GGUCUGGGCGCAGCGCAAGCUGACGGUACAGGCC
	RNAfold	((((((..((((((....)))).))...))))))
	Mfold	((((((..((((((....)))).))...))))))
	AveRNA	((((((..((((((....)))).))...))))))
miRmR_348	mRNA-miRNA sequence	GGGAGAGGGUUUAAUCAGUACGAAAGUACGGAUUGGAUCCGCAAGG
	RNAfold	......((((((((((.((((....)))).))))))))))......
	Mfold	......((((((((((.((((....)))).))))))))))......
	AveRNA	......((((((((((.((((....)))).))))))))))......

S-Table 4. Comparison of RNA secondary structure with different placeholders inserted in the binding site of mRNA-miRNA sequence.

RNA_ID	item	mRNA-miRNA sequence and predicted secondary structures
miRmR_16	mRNA-miRNA sequence	GGCGUCGCACCUUCGGGUGAAGUCGCC
	0 placeholder	((((...((((....))))....))))
	2 placeholders	((((...((((....))))....))))
	5 placeholders	((((...((((....))))....))))
miRmR_28	mRNA-miRNA sequence	GGGCCCGCCGCGCCAUGCCUGUGGCCGGCCC
	0 placeholder	(((((.((((((.......)))))).)))))
	2 placeholders	(((((.((((((.......)))))).)))))
	3 placeholders	(((((.((((((.......)))))).)))))
miRmR_64	mRNA-miRNA sequence	GAGGGUGGAACCGCGCUUCGGCGUCCCUC
	0 placeholder	(((((.......((((....)))))))))
	3 placeholders	(((((.......((((....)))))))))
	4 placeholders	(((((.......((((....)))))))))
miRmR_50	mRNA-miRNA sequence	GGUCUGGGCGCAGCGCAAGCUGACGGUACAGGCC
	0 placeholder	((((((..((((((....)))).))...))))))
	1 placeholder	((((((..((((((....)))).))...))))))
	3 placeholders	((((((..((((((....)))).))...))))))
miRmR_291	mRNA-miRNA sequence	GGGAGAGGGUUUAAUAUAUACGAAAGUAGUCAUUGGAUCCGCAAGG
	0 placeholder	......(((((((((...(((....)))...)))))))))......
	2 placeholders	......(((((((((...(((....)))...)))))))))......
	4 placeholders	......(((((((((...(((....)))...)))))))))......

S-Table 5. The data for different algorithms and fingerprints with accuracy and roc_auc.

Model	accuracy				roc_auc			
	morgan		rdkit		morgan		rdkit	
	Mean	STD	Mean	STD	Mean	STD	Mean	STD
LR	0.7316	0.0211	0.7342	0.0228	0.5138	0.0375	0.5004	0.0364
LDA	0.7290	0.0219	0.7308	0.0202	0.5943	0.0357	0.5996	0.0378
KNN	**0.8214**	**0.0194**	**0.8087**	**0.0184**	**0.8619**	0.0213	0.8447	0.0224
CART	**0.8001**	**0.0169**	0.7907	0.0227	0.7462	**0.0191**	0.7387	0.0295
NB	0.3325	0.0567	0.6724	0.0335	0.4579	0.0355	0.5877	0.0465
SVM	0.7525	0.0206	0.7671	**0.0188**	0.7385	0.0398	0.7394	0.0429
RF	**0.8413**	**0.0187**	**0.8383**	**0.0182**	**0.8702**	0.0253	**0.8731**	0.0217

S-Table 6. The 20 predicted miRNA-mRNA interactions.

	RNA_ID	Score	Predicted probably
1	miRmR_328	0.810833	1
2	miRmR_339	0.806667	1
3	miRmR _347	0.764167	1
4	miRmR _321	0.762500	1
5	miRmR _348	0.750833	1
6	miRmR _337	0.741667	1
7	miRmR _338	0.738167	1
8	miRmR _48	0.725000	1
9	miRmR _346	0.710000	1
10	miRmR _325	0.705667	1
11	miRmR _345	0.667917	1
12	miRmR _336	0.624333	1
13	miRmR _64	0.592500	1
14	miRmR _50	0.590000	1
15	miRmR _23	0.526667	1
16	miRmR _65	0.522500	1
17	miRmR _25	0.505000	1
18	miRmR _57	0.492500	0
19	miRmR _1	0.465000	0
20	miRmR _26	0.457500	0
